# Accelerometry-assessed sleep clusters and obesity in adolescents and young adults: a longitudinal analysis in GINIplus/LISA birth cohorts

**DOI:** 10.1007/s12519-024-00872-5

**Published:** 2025-01-04

**Authors:** Mingming Wang, Claudia Flexeder, Carla P. Harris, Sara Kress, Tamara Schikowski, Annette Peters, Marie Standl

**Affiliations:** 1 Institute of Epidemiology, Helmholtz Zentrum München–German Research Center for Environmental Health, Ingolstädter Landstraße 1, 85764 Neuherberg, Germany; 2 Institute for Medical Information Processing, Biometry and Epidemiology (IBE), Faculty of Medicine, LMU Munich, Pettenkofer School of Public Health, Munich, Germany; 3 Institute and Clinic for Occupational, Social and Environmental Medicine, University Hospital, LMU Munich, Munich, Germany; 4 Department of Pediatrics, Dr. Von Hauner Children’s Hospital, LMU University Hospitals, Munich, Germany; 5 IUF–Leibniz Research Institute for Environmental Medicine, Düsseldorf, Germany; 6 Chair of Epidemiology, Ludwig Maximilians University of Munich, Munich, Germany; 7 German Center for Child and Adolescent Health (DZKJ), Partner Site Munich, Munich, Germany

**Keywords:** Accelerometry, Adolescents, Obesity, Sleep clusters, Young adults

## Abstract

**Background:**

Some studies have revealed various sleep patterns in adolescents and adults using multidimensional objective sleep parameters. However, it remains unknown whether these patterns are consistent from adolescence to young adulthood and how they relate to long-term obesity.

**Methods:**

Seven-day accelerometry was conducted in German Infant Study on the influence of Nutrition Intervention PLUS environmental and genetic influences on allergy development (GINIplus) and Influence of Lifestyle factors on the development of the Immune System and Allergies in East and West Germany (LISA) birth cohorts during the 15-year and 20-year follow-ups, respectively. Five sleep clusters were identified by k-means cluster analysis using 12 sleep characteristics at each follow-up. Adjusted linear and logistic regression models using generalized estimating equations were examined. Further, the interaction effects with time of follow-ups and polygenic risk scores (PRS) for body mass index (BMI) were tested.

**Results:**

Five sleep clusters were classified consistently in both adolescence (*n* = 1347, aged 14.3–16.4 years) and young adulthood (*n* = 1262, aged 19.5–22.4 years). Adolescents in the “good sleep”, “delayed sleep phase”, and “fragmented sleep” clusters displayed greater stability transitioning into young adulthood, while those in the “sleep irregularity and variability”, and “prolonged sleep latency” clusters showed lower stability (*n* = 636). Compared to the “good sleep” cluster, the “prolonged sleep latency” cluster exhibited associations with higher BMI [*β* = 0.56, 95% confidence interval (CI) = (0.06, 1.05)] and increased odds of overweight/obesity [Odds ratio = 1.55, 95% CI = (1.02, 2.34)]. No significant PRS-sleep cluster interaction was found for BMI or overweight/obesity. Among males only, the “delayed sleep phase”, “sleep irregularity and variability” and “fragmented sleep” clusters showed stronger associations with overweight/obesity as age increased.

**Conclusion:**

Adolescents and young adults shared five consistent sleep patterns, with the “prolonged sleep latency” pattern linked to higher BMI and overweight/obesity.

**Graphical abstract:**

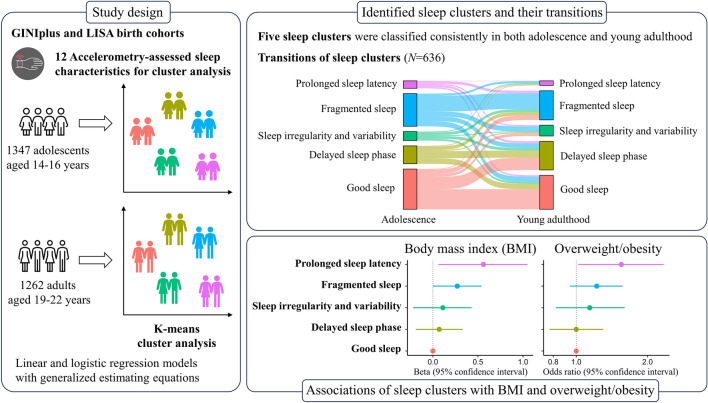

**Supplementary Information:**

The online version contains supplementary material available at 10.1007/s12519-024-00872-5.

## Introduction

Insufficient sleep is associated with increased risks of obesity and cardiovascular diseases [1, 2], prompting its inclusion as an eighth essential factor for cardiovascular health by the American Heart Association in 2022 [3]. Apart from sleep duration [4], other sleep dimensions are recognized as obesity risk factors, such as sleep efficiency [5], variability [6], and timing [7]. These sleep characteristics are interrelated within an individual, making sleep health a multidimensional construct with overlapping components [8].

Some studies have identified diverse sleep patterns in children, adolescents, and adults, using comprehensive approaches by considering multiple objective sleep characteristics [9–13]. Cluster analysis effectively groups similar individuals by accounting for the correlations among various sleep characteristics [8]. Recently, we identified five sleep clusters in German adolescents by K-means cluster analysis across 12 accelerometry-assessed sleep characteristics, and observed an association between “prolonged sleep latency” cluster and higher fat mass index [14]. Another recent study identified three sleep clusters for males and females among Brazil young adults using K-means cluster analysis on seven sleep characteristics measured mainly by accelerometry, with “healthy sleepers” cluster showing lower prevalence of overweight [13]. However, it is poorly understood whether objective sleep patterns change during the transition from adolescence into young adulthood due to substantial physiological, psychological, and environmental shifts [15, 16]. To our knowledge, no study has comprehensively identified sleep patterns with multiple objective sleep characteristics, across both adolescence and young adulthood.

In addition, it is unclear whether objective sleep patterns interact with obesity-related genetic variants [17]. One study reported uncorrected interactions between objective sleep duration and three gene loci affecting body mass index (BMI) in 643 New Zealand children [18]. Furthermore, evidence remains scarce on examining sex differences in relationships between objective sleep characteristics and obesity during the transition from adolescence into young adulthood. A study from USA revealed that subjective short sleep was linked to obesity only in adolescent males, but was linked to incident obesity in both sexes during young adulthood [19].

Therefore, we aimed to investigate longitudinal associations of clustering-identified sleep patterns, using multidimensional accelerometry-assessed sleep characteristics, with BMI and overweight/obesity in adolescence and young adulthood, and to explore sleep interaction effects with time of follow-ups and genetic risk.

## Methods

### Study population

Data were obtained from two ongoing German birth cohorts, German Infant Study on the influence of Nutrition Intervention PLUS environmental and genetic influences on allergy development (GINIplus) and Influence of Lifestyle factors on the development of the Immune System and Allergies in East and West Germany (LISA). More details are available elsewhere [20–22]. For two cohorts, 1682 participants from Munich and Wesel at the 15-year follow-up (15 y) between 2011 and 2014, and 1595 participants from Munich, Wesel and Bad Honnef at the 20-year follow-up (20 y) between 2016 and 2020, consented to and completed accelerometry measurements.

**Fig. 1:**
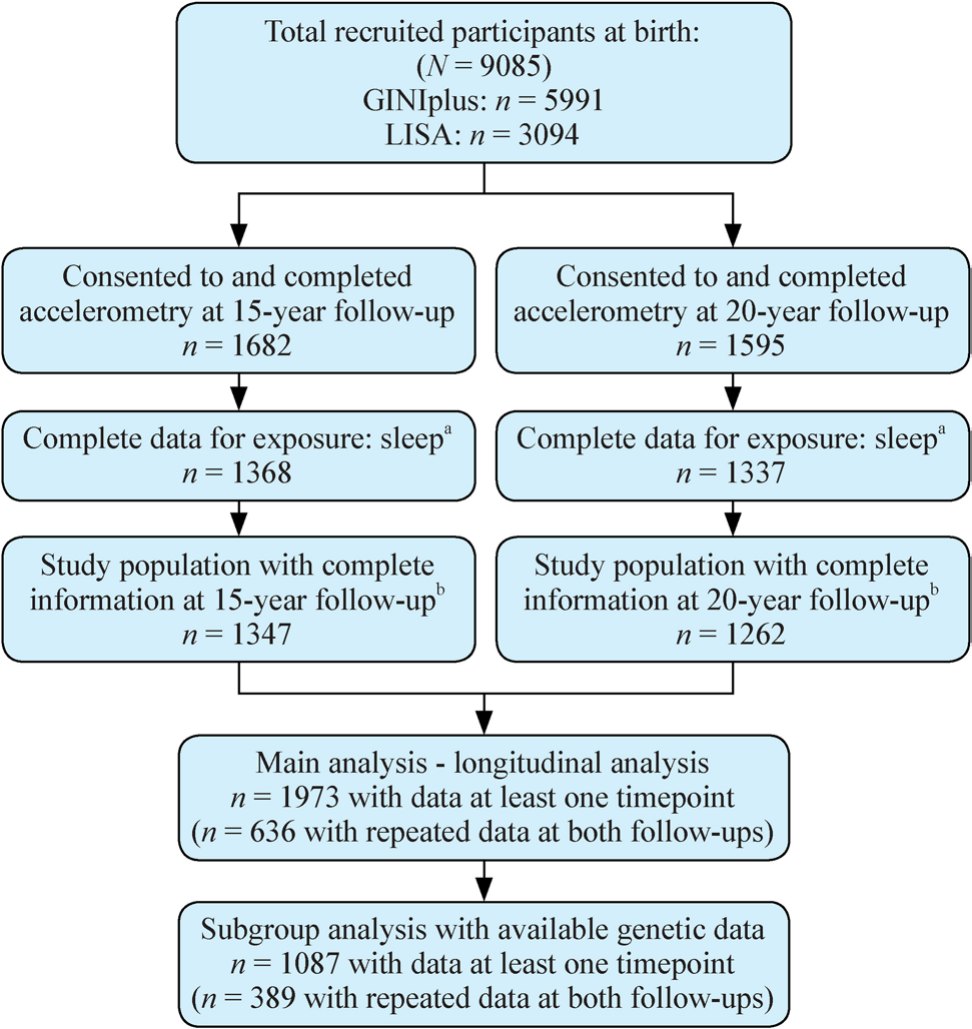
Flow chart of participants. *GINIplus* German Infant Study on the influence of Nutrition Intervention PLUS environmental and genetic influences on allergy development, *LISA* Influence of Lifestyle factors on the development of the Immune System and Allergies in East and West Germany. **a**: Inclusion for sleep data: (1) at least three weekdays and one weekend day of valid accelerometry recording for ≥ 10 hours/day; (2) 2 hours ≤ daily total sleep time ≤ 15 hours; (3) daily sleep efficiency ≥ 20%; (4) daily sleep onset timing between 17:00 and 08:00. **b**: Inclusion for participants: (1) participants in Munich and Wesel study centers; (2) complete outcomes; (3) complete confounders, except for total energy intake

For the main analyses, a total of 1973 participants at 15 y (*n* = 1347) and/or 20 y (*n* = 1262) in Munich or Wesel were included, with 636 participants having repeated data at both follow-ups. A subset of 1087 participants (*n* = 775 at 15 y; *n* = 701 at 20 y) with genetic data were used for genetic analysis, including 389 with repeated exposures and outcomes. Participants were included if they had at least three weekdays and one weekend day of valid accelerometry data for ≥ 10 hours/day. Figure 1 offers detailed inclusion criteria. Local ethics committees approved both cohorts, and all participants and their parents wrote informed consents.

### Sleep assessment, characteristics, and clusters

#### Accelerometry

A validated triaxial accelerometer (ActiGraph GT3X, Pensacola, Florida) was applied at both follow-ups [23, 24]. Participants wore accelerometers on non-dominant hand-side wrists at night for assessing sleep, and on dominant hips during the day for measuring physical activity (PA), for seven consecutive days during a regular school/work week. Participants also kept sleep diaries to record the times they went to bed and got up, corresponding with the transition of the accelerometer from hip to wrist in the evening and back in the morning. Accelerometry measurements details are available elsewhere [25–27].

#### Objective sleep characteristics

Accelerometry-assessed sleep data were analyzed with ActiLife software (firmware 4.4.0; version 5.5.5 at 15 y; version 6.11.2 at 20 y) based on the Sadeh algorithm [28]. Accelerations were sampled at 30 Hz, converted to proprietary “activity count units”, and stored at 1 Hz, aggregated over one-second intervals. The “probability of sleep” was calculated as a score centered around zero for each minute of recorded time-in-bed. A score was classified as “asleep” if it was zero or positive, and “awake” if negative. Six sleep characteristics were generated per valid night: (1) total sleep time (hours), algorithm-scored total “asleep” minutes divided by 60; (2) sleep efficiency (%), ratio of algorithm-scored “asleep” minutes to diary-recorded total time in bed; (3) sleep midpoint timing (24-hour clock), first algorithm-scored minute of “asleep” plus half of total sleep time, converted to 24-hour clock; (4) sleep latency (minutes), total number of minutes between diary-recorded timing of going to bed and the first algorithm-scored minute of “asleep”; (5) time awake per hour after sleep onset (minutes/hour), total algorithm-scored awake minutes after sleep onset, divided by hours spent in bed after sleep onset; and (6) awakenings per hour after sleep onset (numbers/hour), total algorithm-scored different awakening episodes after sleep onset, divided by hours spent in bed after sleep onset. Six average sleep characteristics were computed as mean value across all valid days, and their day-to-day variability was computed as standard deviation (SD). Twelve sleep characteristics were included for cluster analysis: (averaged) total sleep time (TST), sleep efficiency (SE), sleep latency (SL), time awake per hour after sleep onset (WASO/h), awakenings per hour after sleep onset (awakenings/h), sleep midpoint timing (SMT), SD in total sleep time (SD in TST), SD in sleep efficiency (SD in SE), SD in sleep latency (SD in SL), SD in time awake per hour after sleep onset (SD in WASO/h), and SD in awakenings per hour after sleep onset (SD in awakenings/h), SD in sleep midpoint timing (SD in SMT).

#### Sleep clusters

Five sleep clusters at 15 y were identified previously: good sleep, delayed sleep phase, sleep irregularity and variability, fragmented sleep, and prolonged sleep latency [14]. Sleep cluster at 20 y were identified in the current study.

Subjective sleep characteristics included reported time in bed, sleep quality, sleep difficulties, difficulty falling asleep, difficulty staying asleep. More detailed definitions are presented in Supplementary Method S1.

### Body mass index measurements

At 15 y, participants’ body weight (kg) and height (m) were measured objectively by physical examinations (*n* = 1197) and parent-reported by questionnaires (*n* = 150). At 20 y, participants self-reported weight and height by questionnaires. BMI was calculated as weight divided by height squared (kg/m^2^). The objective BMI strongly correlated with subjective BMI (Pearson coefficient = 0.95) at 15 y, and BMI z-scores were computed following World Health Organization references [29]. BMI were categorized into overweight/obesity and non-overweight/obesity, defined as BMI z-scores > 1 and ≤ 1 for adolescents [29], and BMI ≥ 25 and < 25 kg/m^2^ for adults [30], respectively.

### Confounders

Potential confounders included time of follow-ups (15 y and 20 y), the time-independent variables: sex, study (GINI observation arm, GINI intervention arm, and LISA study), study center (Munich and Wesel), parental highest education level (low/medium: ≤ 10th grade; high: > 10th grade); and the time-dependent variables: age at BMI measurements, BMI measurement methods (examination vs. questionnaire at 15 y; questionnaire at 20 y), season of sleep measurements (spring, summer, autumn, and winter), accelerometry-measured sedentary behavior, moderate-to-vigorous physical activity (MVPA), and total energy intake (kcal) at two follow-ups, respectively. In both follow-ups, sedentary, MVPA [25] and total energy intake were obtained using the same protocol. Accelerometry-measured PA was categorized into sedentary (by Aguilar-Farías [31]), light, moderate, and vigorous PA using triaxial cutoffs by Romanzini [32], with moderate and vigorous PA combined into MVPA. This study used the averaged sedentary (hours) and MVPA (minutes) from all valid days. Total energy intake was computed from a self-administered food frequency questionnaire [33, 34], with missing values (*n* = 284 at 15 y; *n* = 243 at 20 y) imputed using a linear regression model based on sex, age, study, study center, and parental highest education [35].

### Polygenic risk score for BMI

Genotyping for GINIplus and LISA was conducted using Affymetrix Chip 5.0 and 6.0 (Thermo Fisher, USA) in Munich and Infinium Global Screening Array GSA v2 MD (Illumina, USA) in Wesel. Quality control and genotype imputation details were previously published [36, 37]. PRS for BMI were computed according to 97 genome-wide significant single nucleotide polymorphisms (SNPs) [38]. Of the 97 SNPs, 95 SNPs available in Munich, and 96 SNPs available in Wesel were included for polygenic risk score (PRS) calculation, respectively [39]. Standardized PRS were used, with higher values indicating increased risk for high BMI. SNPs lists can be found previously [39].

### Statistical analysis

At both follow-ups, 12 sleep characteristics were first standardized, then tested for their Spearman correlation, followed by hierarchical cluster analysis using Euclidean distance and Ward’s linkage (Ward.D2), and k-means cluster analysis. Details on sleep cluster number selection at 15 y has been described previously [14]. The final number of sleep clusters at 20 y was determined to be five after evaluating: (1) interpretation of k-means results; (2) results of principal component analysis (Supplementary Table S1, where five components balance the criteria of eigenvalues > 1 and a cumulative variance > 80%); (3) dendrogram of hierarchical clustering (Supplementary Fig. S1); and (4) visualized results from sum of squares method (Supplementary Fig. S2). The principal component analysis plots (Supplementary Fig. S3) visually validated similar clustering patterns at 15 y and 20 y, according to a systematic framework [40]. In the final K-means cluster analysis at each follow-up, five clusters were designated, using 50 random initial centroids. In addition, the K-means cluster analyses were limited to participants with data available at both time points (*n* = 636) to test the agreement of sleep pattern classification between the full dataset and the subsample. Differences in characteristics by sex and by sleep clusters were assessed using one-way analysis of variance and Kruskal–Wallis rank sum test for continuous variables, and Chi-square test for categorical variables, followed by Bonferroni-adjusted post-hoc tests.

Linear and logistic regression models using generalized estimating equations (GEE) were used to evaluate longitudinal associations of sleep clusters with BMI and overweight/obesity. GEE models can estimate population-averaged effects across repeated measurements and provide robust estimates, even with only one time-point data for some participants. Boxplot inspection identified BMI outliers, none of which were excluded. Three models were examined: Model 1 was adjusted for time of follow-ups, sex, age, study, study center, parental highest education, and BMI measurement methods; Model 2 was further adjusted for season, sedentary, MVPA, and total energy intake; Model 3 was Model 2 plus PRS interaction term with sleep clusters. The results were presented as *β* with 95% confidence interval (CI), and odds ratio (OR) with 95%CI, respectively. *P* < 0.05 was considered statistically significant.

Furthermore, the interaction effects of sleep clusters with sex and time of follow-ups were examined, followed by sex- and time-stratified analyses (cross-sectional analyses at two time-points). Four sensitivity analyses were conducted: (1) including participants with repeated sleep and BMI data at both follow-ups (*n* = 636); (2) excluding participants with missing total energy intake; (3) excluding participants with parent-reported BMI at 15 y; (4) determining sleep clusters by sleep characteristics excluding weekends (the nights from Friday to Saturday and Saturday to Sunday). All statistical analyses were conducted in *R* (version 4.3.1) [41].

## Results

Table 1 presents participants characteristics overall and by sex, in adolescence and young adulthood. In adolescence, males had higher prevalence of having overweight/obesity than females, yet no significant difference between sexes was observed in young adulthood. From adolescence to young adulthood, averaged TST decreased (7.2–6.6 hours), SE increased (79.3%–84.4%), SMT was one hour later (2:36–3:36) and SL was shortened (18.7–6.8 minutes, Table 2). Day-to-day variability in six sleep characteristics exhibited minimal changes, except for a decrease of SD in SL (14.9–6.7 minutes). In addition, females had higher TST than males (7.3 vs. 7.0 hours in adolescence; 6.8 vs. 6.5 hours in young adulthood). More details can be found in Supplementary Table S2.

**Table 1:** Participants characteristics in adolescence and young adulthood

Characteristics	Adolescence				Young adulthood			
	Total	Male	Female	*P* value	Total	Male	Female	*P* value
*N*	1347	611	736		1262	491	771	
Age, y	15.2 ± 0.3	15.2 ± 0.3	15.2 ± 0.3	0.397	20.2 ± 0.4	20.3 ± 0.4	20.2 ± 0.4	** < 0.001**
Study, *n* (%)				0.238				**0.036**
GINIplus observation	497 (36.9)	217 (35.5)	280 (38.0)		488 (38.7)	174 (35.4)	314 (40.7)	
GINIplus intervention	509 (37.8)	226 (37.0)	283 (38.5)		480 (38.0)	185 (37.7)	295 (38.3)	
LISA	341 (25.3)	168 (27.5)	173 (23.5)		294 (23.3)	132 (26.9)	162 (21.0)	
Study center, *n* (%)				**0.016**				**0.003**
Munich	818 (60.7)	393 (64.3)	425 (57.7)		762 (60.4)	322 (65.6)	440 (57.1)	
Wesel	529 (39.3)	218 (35.7)	311 (42.3)		500 (39.6)	169 (34.4)	331 (42.9)	
Weight, kg	61.1 ± 11.0	63.9 ± 11.8	58.7 ± 9.7	** < 0.001**	68.7 ± 12.6	76.6 ± 10.9	63.8 ± 11.0	** < 0.001**
Height, cm	171.4 ± 8.1	176.2 ± 7.4	167.4 ± 6.3	** < 0.001**	174.5 ± 9.6	183.2 ± 6.8	169.0 ± 6.4	** < 0.001**
BMI, kg/m^2^	20.7 ± 3.0	20.5 ± 3.1	20.9 ± 3.0	**0.022**	22.5 ± 3.3	22.8 ± 2.9	22.3 ± 3.5	**0.009**
BMI z-score	0.1 ± 1.0	0.0 ± 1.1	0.1 ± 0.9	0.661				
Overweight/obesity, *n* (%)				**0.004**				0.635
No	1120 (83.1)	488 (79.9)	632 (85.9)		1040 (82.4)	401 (81.7)	639 (82.9)	
Yes	227 (16.9)	123 (20.1)	104 (14.1)		222 (17.6)	90 (18.3)	132 (17.1)	
BMI measurement methods, *n* (%)				0.342				NA
Examination	1197 (88.9)	537 (87.9)	660 (89.7)					
Questionnaire	150 (11.1)	74 (12.1)	76 (10.3)		1262 (100.0)	491 (100.0)	771 (100.0)	
Sleep clusters, *n* (%)				** < 0.001**				** < 0.001**
Good sleep	440 (32.7)	154 (25.2)	286 (38.8)		389 (30.8)	103 (21.0)	286 (37.1)	
Delayed sleep phase	245 (18.2)	101 (16.5)	144 (19.6)		330 (26.2)	133 (27.1)	197 (25.6)	
Sleep irregularity and variability	130 (9.6)	54 (8.8)	76 (10.3)		132 (10.5)	60 (12.2)	72 (9.3)	
Fragmented sleep	424 (31.5)	249 (40.8)	175 (23.8)		340 (26.9)	158 (32.2)	182 (23.6)	
Prolonged sleep latency	108 (8.0)	53 (8.7)	55 (7.5)		71 (5.6)	37 (7.5)	34 (4.4)	
Season, *n *(%)				0.326				0.454
Spring	355 (26.4)	168 (27.5)	187 (25.4)		349 (27.7)	129 (26.3)	220 (28.5)	
Summer	198 (14.7)	79 (12.9)	119 (16.2)		342 (27.1)	129 (26.3)	213 (27.6)	
Autumn	437 (32.4)	205 (33.6)	232 (31.5)		297 (23.5)	127 (25.9)	170 (22.1)	
Winter	357 (26.5)	159 (26.0)	198 (26.9)		274 (21.7)	106 (21.6)	168 (21.8)	
Total energy intake, kcal/day	2076.4 ± 647.6	2374.5 ± 645.0	1851.5 ± 552.1	** < 0.001**	1776.3 ± 661.1	2146.2 ± 690.7	1583.6 ± 555.3	** < 0.001**
Sedentary behavior, hours	8.3 ± 1.4	8.0 ± 1.5	8.5 ± 1.3	** < 0.001**	8.4 ± 1.5	8.3 ± 1.7	8.4 ± 1.5	0.762
MVPA, minutes	50.8 ± 27.1	57.7 ± 27.3	45.2 ± 25.7	** < 0.001**	46.2 ± 23.7	48.9 ± 23.6	44.6 ± 23.6	**0.002**
Parental highest education, *n* (%)				0.748				**0.017**
Low/medium	395 (29.3)	176 (28.8)	219 (29.8)		355 (28.1)	119 (24.2)	236 (30.6)	
High	952 (70.7)	435 (71.2)	517 (70.2)		907 (71.9)	372 (75.8)	535 (69.4)	

The results are presented as mean ± standard deviation or *n* (%). *BMI* body mass index, *MVPA* moderate-to-vigorous physical activity, *GINIplus* German Infant Study on the influence of Nutrition Intervention PLUS environmental and genetic influences on allergy development; *LISA* Influence of Lifestyle factors on the development of the Immune System and Allergies in East and West Germany

Overweight/obesity: BMI z-score > 1 for adolescents; BMI ≥ 25 kg/m^2^ for adults according to World Health Organization

The number of participants with available total energy intake: *n* = 1063 in adolescence; *n* = 1019 in young adulthood. *P* value: one-way analysis of variance was used for continuous variables, and Chi-square test was used for categorical variables. *P* values < 0.05 were highlighted in bold

**Table 2:** Sleep characteristics in the total population and in five sleep clusters during adolescence and young adulthood

Sleep characteristics	Total		Sleep clusters										
			Good sleep		Delayed sleep phase		Sleep irregularity and variability		Fragmented sleep		Prolonged sleep latency		*p value
	Mean	Median (IQR)	Mean	Median (IQR)	Mean	Median (IQR)	Mean	Median (IQR)	Mean	Median (IQR)	Mean	Median (IQR)	
**Adolescence** **(14–16** **y),** ***n*** **(%)**	**1347**		**440 (32.7)**		**245 (18.2)**		**130 (9.6)**		**424 (31.5)**		**108 (8.0)**		
Averages across all valid days													
TST, h	7.2	7.2 (0.9)	7.5	7.5 (0.7)^a^	7.2	7.2 (0.9)^b^	6.9	6.8 (0.8)^c^	6.9	6.9 (0.7)^c^	7.0	7.0 (0.9)^c^	< 0.001
SE, %	79.3	79.8 (8.0)	84.4	84.2 (4.2)^a^	82.4	82.3 (5.3)^b^	74.8	75.7 (7.0)^cd^	75.5	76.1 (5.3)^c^	72.6	73.2 (6.9)^d^	< 0.001
SL, min	18.7	15.9 (13.4)	14.1	12.3 (9.9)^a^	16.5	14.7 (11.8)^b^	18.9	17.6 (11.3)^b^	17.8	16.8 (10.8)^b^	45.4	42.4 (17.2)^c^	< 0.001
WASO/h, min/h	10.6	10.2 (4.5)	7.9	8.0 (2.5)^a^	8.9	9.0 (2.5)^b^	13.4	12.9 (4.1)^c^	13.1	12.8 (3.1)^c^	12.6	12.2 (4.0)^c^	< 0.001
Awakenings/h, numbers/h	2.9	2.9 (0.8)	2.5	2.6 (0.7)^a^	2.6	2.6 (0.7)^a^	2.8	2.8 (0.6)^b^	3.4	3.3 (0.6)^c^	2.9	2.9 (0.7)^b^	< 0.001
SMT, 24-h clock	2:36	2:36 (54 min)	2:36	2:30 (42 min)^a^	3:18	3:18 (48 min)^b^	2:30	2:30 (48 min)^a^	2:12	2:12 (42 min)^c^	2:30	2:36 (54 min)^a^	< 0.001
Day-to-day variability across all valid days													
SD in TST, min	61.4	57.6 (38.1)	51.0	50.1 (28.8)^a^	84.5	81.3 (38.8)^b^	86.8	84.9 (39.8)^b^	49.6	48.2 (27.6)^a^	66.9	59.7 (38.3)^c^	< 0.001
SD in SE, %	5.6	5.0 (3.0)	4.2	4.0 (2.1)^a^	5.1	4.9 (2.4)^b^	11.1	10.7 (3.8)^c^	5.1	5.0 (2.4)^b^	7.5	7.2 (2.9)^d^	< 0.001
SD in SL, min	14.9	11.7 (12.2)	10.3	8.5 (7.9)^a^	12.5	10.8 (10.1)^b^	16.5	15.1 (13.7)^c^	13.5	12.3 (9.9)^bc^	42.7	38.7 (18.8)^d^	< 0.001
SD in WASO/h, min/h	3.3	3.0 (1.9)	2.5	2.4 (1.3)^a^	3.0	2.9 (1.6)^b^	6.8	6.4 (2.1)^c^	3.1	3.0 (1.5)^b^	4.3	4.1 (2.1)^d^	< 0.001
SD in Awakenings/h, numbers/h	0.6	0.5 (0.3)	0.5	0.5 (0.2)^a^	0.6	0.6 (0.3)^b^	0.7	0.6 (0.4)^b^	0.5	0.5 (0.2)^ac^	0.6	0.6 (0.3)^bc^	< 0.001
SD in SMT, min	67.5	64.4 (35.8)	56.3	55.4 (26.3)^a^	96.1	92.9 (37.3)^b^	75.0	73.9 (39.3)^c^	59.3	59.3 (31.1)^a^	71.3	67.3 (37.3)^c^	< 0.001
**Young adulthood (19–22 y),** ***n*** **(%)**	**1262**		**389 (30.8)**		**330 (26.2)**		**132 (10.5)**		**340 (26.9)**		**71 (5.6)**		
Averages across all valid days													
TST, h	6.6	6.6 (1.0)	7.1	7.1 (0.8)^a^	6.7	6.7 (0.9)^b^	6.3	6.3 (0.8)^c^	6.4	6.4 (0.9)^c^	6.3	6.2 (1.0)^c^	< 0.001
SE, %	84.4	85.2 (7.5)	88.4	88.3 (4.1)^a^	87.7	87.7 (4.3)^a^	80.0	80.5 (6.1)^b^	79.8	80.4 (4.7)^b^	76.8	78.6 (7.7)^b^	< 0.001
SL, min	6.8	5.2 (5.8)	5.0	4.2 (4.3)^a^	5.4	4.6 (4.5)^a^	7.6	7.6 (6.2)^b^	6.6	6.0 (5.6)^b^	22.4	21.1 (8.5)^c^	< 0.001
WASO/h, min/h	8.6	8.2 (4.3)	6.4	6.4 (2.5)^a^	6.8	6.9 (2.5)^a^	11.2	10.9 (3.3)^b^	11.5	11.1 (2.8)^b^	11.6	10.8 (5.4)^b^	< 0.001
Awakenings/h, numbers/h	2.9	2.9 (1.0)	2.5	2.6 (0.7)^a^	2.5	2.5 (0.8)^a^	2.9	2.9 (0.8)^b^	3.6	3.5 (0.6)^c^	3.1	3.1 (0.9)^b^	< 0.001
SMT, 24-h clock	3:36	3:30 (84 min)	3:24	3:18 (78 min)^a^	4:06	4:00 (84 min)^b^	3:42	3:36 (78 min)^c^	3:18	3:12 (66 min)^a^	3:42	3:30 (72 min)^ac^	< 0.001
Day-to-day variability across all valid days													
SD in TST, min	64.8	61.1 (36.5)	50.2	48.2 (27.0)^a^	81.6	79.6 (35.1)^b^	84.3	81.4 (38.4)^b^	56.3	53.4 (28.9)^c^	70.9	62.3 (32.6)^d^	< 0.001
SD in SE, %	4.9	4.4 (2.7)	3.4	3.1 (1.7)^a^	4.3	4.1 (1.9)^b^	9.9	9.0 (2.9)^c^	5.0	4.9 (2.0)^d^	6.7	6.0 (3.4)^e^	< 0.001
SD in SL, min	6.7	5.2 (5.0)	4.7	4.0 (3.3)^a^	5.3	4.6 (4.3)^a^	8.8	8.1 (8.2)^b^	6.3	5.7 (4.3)^c^	22.8	19.9 (7.7)^d^	< 0.001
SD in WASO/h, min/h	2.9	2.5 (1.5)	2.0	1.9 (1.0)^a^	2.5	2.4 (1.1)^b^	5.8	5.3 (1.7)^c^	3.0	3.0 (1.3)^d^	3.3	3.1 (1.7)^d^	< 0.001
SD in Awakenings/h, numbers/h	0.6	0.6 (0.3)	0.5	0.5 (0.2)^a^	0.7	0.6 (0.3)^b^	0.9	0.9 (0.4)^c^	0.6	0.6 (0.3)^b^	0.7	0.6 (0.3)^b^	< 0.001
SD in SMT, min	70.4	65.2 (46.0)	49.9	47.9 (29.2)^a^	94.5	90.8 (41.8) ^b^	93.0	88.3 (53.0)^b^	61.9	57.7 (37.4)^c^	69.5	65.1 (42.9)^c^	< 0.001

The results are presented as mean and median (IQR) of sleep characteristics, given that most of them did not follow a normal distribution. *P* value: Kruskal–Wallis test with Dunn post hoc tests and Bonferroni adjustment. Sharing the same letter (a b c d e) are considered not significantly different (adjusted *P* values < 0.05). *Awakenings/h* awakenings per hour after sleep onset, *IQR* interquartile range, *SD* standard deviation, *SE* sleep efficiency, *SL* sleep latency, *SMT* sleep midpoint timing, *TST* total sleep time, *WASO/h* time awake per hour after sleep onset

**Fig. 2:**
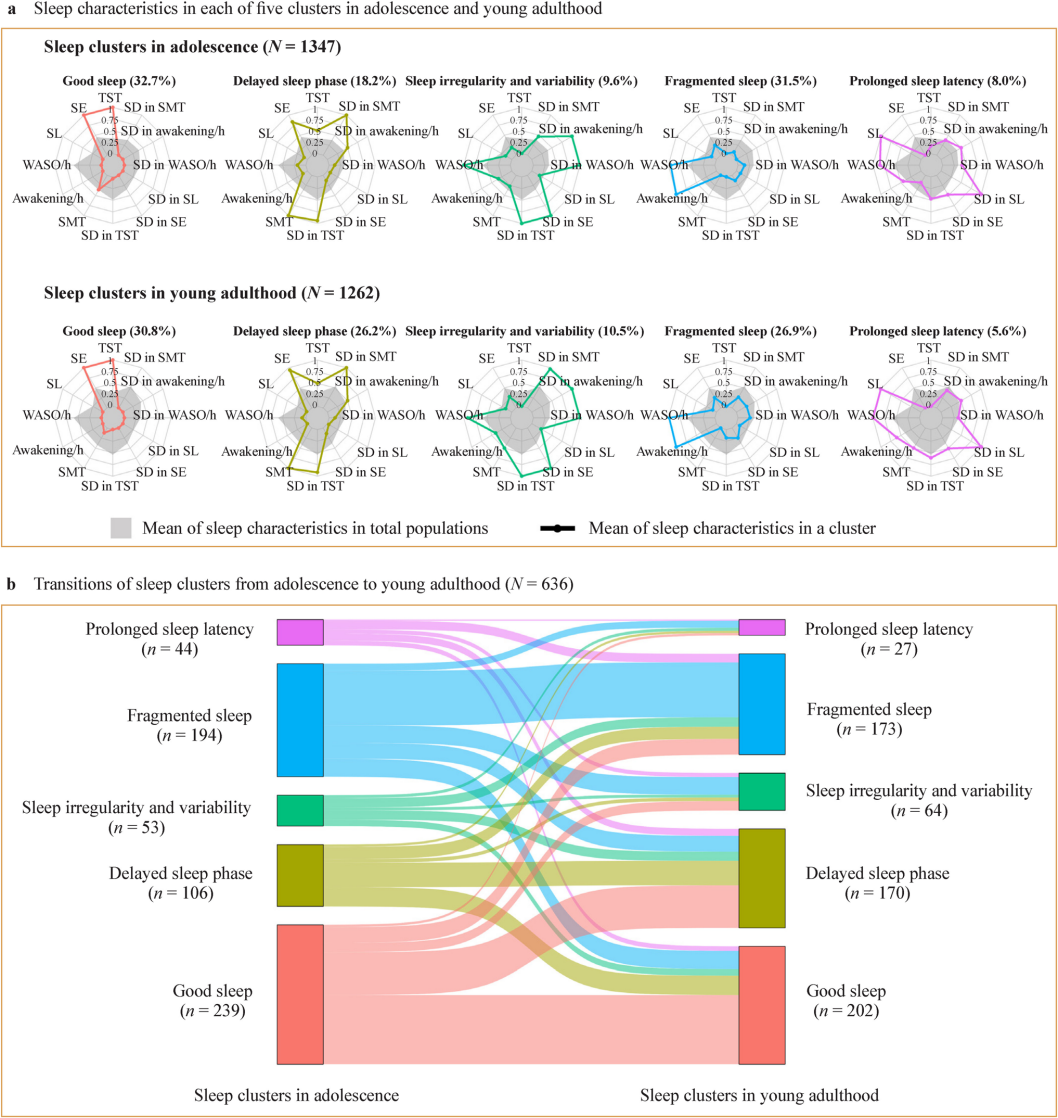
Five sleep clusters and their transitions in adolescence and young adulthood. **a**, Sleep characteristics in each of five clusters in adolescence and young adulthood. The 12 axes represent 12 sleep characteristics, which were scaled to the value between 0 and 1. Five colors represent five sleep clusters (same as b). *Awakenings/h* awakenings per hour after sleep onset, *SD* standard deviation, *SE* sleep efficiency, *SL* sleep latency, *SMT* sleep midpoint timing, *TST* total sleep time, *WASO/h* time awake per hour after sleep onset. **b**, Transitions of sleep clusters from adolescence to young adulthood (*N* = 636), during 5-year follow-up. The line thickness between adolescence and young adulthood represents the proportions of each sleep cluster in adolescence that remained in the same cluster or transitioned to a different cluster in young adulthood. The thicker the line, the higher the proportion. Modified from Wang M, et al., Obesity (Silver Spring), 2024, under CC BY-NC-ND 4.0

Five sleep clusters in young adulthood were identified and consistent with those during adolescence [14], and named by their unique parameters: (1) “good sleep”, marked by higher TST and SE; (2) “delayed sleep phase”, distinguished by later SMT, higher SE, and SD in SMT; (3) “sleep irregularity and variability”, characterized by higher SD in most sleep characteristics and higher WASO/h; (4) “fragmented sleep”, demonstrating longer WASO/h and more frequent awakenings/h; and (5) “prolonged sleep latency”, exhibiting higher SL, SD in SL, and WASO/h (Table 2 and Fig. 2a). Supplementary Table S3 demonstrates that, in adolescence, the “good sleep” and “delayed sleep phase” clusters subjectively reported shorter time in bed with higher sleep quality, while the “prolonged sleep latency” cluster had the opposite. In young adulthood, the “delayed sleep phase” cluster reported the lowest time in bed. Supplementary Table S2 and Supplementary Table S4 display sex-stratified sleep characteristics and participants characteristics in five sleep clusters. Figure 2b and Supplementary Table S5 illustrate sleep clusters transition from adolescence to young adulthood among participants with repeated data (*n* = 636). Adolescents within the “good sleep”, “delayed sleep phase”, and “fragmented sleep” clusters were more stable into the same cluster during the transition (consistency rate ≥ 40%). Conversely, adolescents within the “sleep irregularity and variability”, and “prolonged sleep latency” clusters showed lower stability (consistency rate < 10%). Similar transition patterns were found in both sexes. Furthermore, the re-identified sleep clusters specifically among participants with repeated data showed strong agreement with the original analysis (Cohen’s kappa = 0.87 in adolescence, 0.78 in young adulthood), supporting the robustness and representativeness of the sleep pattern classifications (Supplementary Table S6).

In GEE regression models, compared to the “good sleep” cluster, the “prolonged sleep latency” cluster was associated with higher BMI [*β* = 0.56, 95% CI = (0.06, 1.05)] and increased odds of having overweight/obesity [OR = 1.55, 95% CI = (1.02, 2.34)], after adjustment for confounders (Model 2, Table 3). In addition, PRS was associated with higher BMI [*β* = 0.65, 95% CI = (0.39, 0.91)] and overweight/obesity [OR = 1.52, 95% CI = (1.15, 2.00)], yet had no significant gene-sleep cluster interaction (Model 3, Table 3). No interaction between sleep clusters and sex was detected (*P*-interaction > 0.05, Supplementary Table S7).

**Table 3:** Associations of sleep clusters and genetic risk with BMI and overweight/obesity

Sleep clusters		Model 1 (2609 observations)		Model 2 (2609 observations)		Model 3 (1476 observations)		
BMI	Observations	*β* (95%CI)	*P* value	*β* (95% CI)	*P* value	*β* (95%CI)	*P* value	*P* value interaction
Good sleep	829	Ref		Ref				
Delayed sleep phase	575	0.07 (− 0.18, 0.33)	0.574	0.07 (−0.19, 0.33)	0.587	0.01 (− 0.35, 0.36)	0.969	0.358
Sleep irregularity and variability	262	0.05 (− 0.27, 0.38)	0.752	0.11 (−0.22, 0.43)	0.528	0.13 (−0.31, 0.57)	0.551	0.606
Fragmented sleep	764	0.22 (− 0.05, 0.48)	0.109	0.27 (−0.00, 0.54)	0.053	0.19 (−0.16, 0.54)	0.283	0.722
Prolonged sleep latency	179	0.47 (− 0.02, 0.97)	0.062	0.56 (0.06, 1.05)	**0.028**	0.78 (0.03, 1.54)	**0.042**	0.228
PRS						0.65 (0.39, 0.91)	** < 0.001**	
Overweight/obesity	Cases/observations	OR (95% CI)	*P* value	OR (95% CI)	*P* value	OR (95% CI)	*P* value	*P* value interaction
Good sleep	15.6%	Ref		Ref				
Delayed sleep phase	16.3%	1.00 (0.77, 1.29)	0.990	1.00 (0.77, 1.30)	0.993	0.98 (0.68, 1.40)	0.904	0.942
Sleep irregularity and variability	19.5%	1.08 (0.78, 1.50)	0.641	1.14 (0.82, 1.60)	0.443	1.43 (0.94, 2.16)	0.095	0.327
Fragmented sleep	17.8%	1.15 (0.89, 1.48)	0.278	1.22 (0.94, 1.57)	0.137	1.40 (0.99, 1.98)	0.055	0.825
Prolonged sleep latency	21.8%	1.41 (0.95, 2.10)	0.087	1.55 (1.02, 2.34)	**0.039**	1.81 (1.01, 3.25)	**0.047**	0.182
PRS						1.52 (1.15, 2.00)	**0.003**	

Model 1: Adjusted for time of follow-ups, sex, age, study, study center, parental highest education, BMI measurement methods;

Model 2: Model 1 + season, sedentary behavior, moderate-to-vigorous physical activity, and total energy intake;

Model 3: Model 2 + PRS interaction term with sleep clusters. *P* values < 0.05 were highlighted in bold

*BMI* body mass index, *CI* confidence interval, *OR* Odds ratio, *PRS* polygenic risk score

**Fig. 3:**
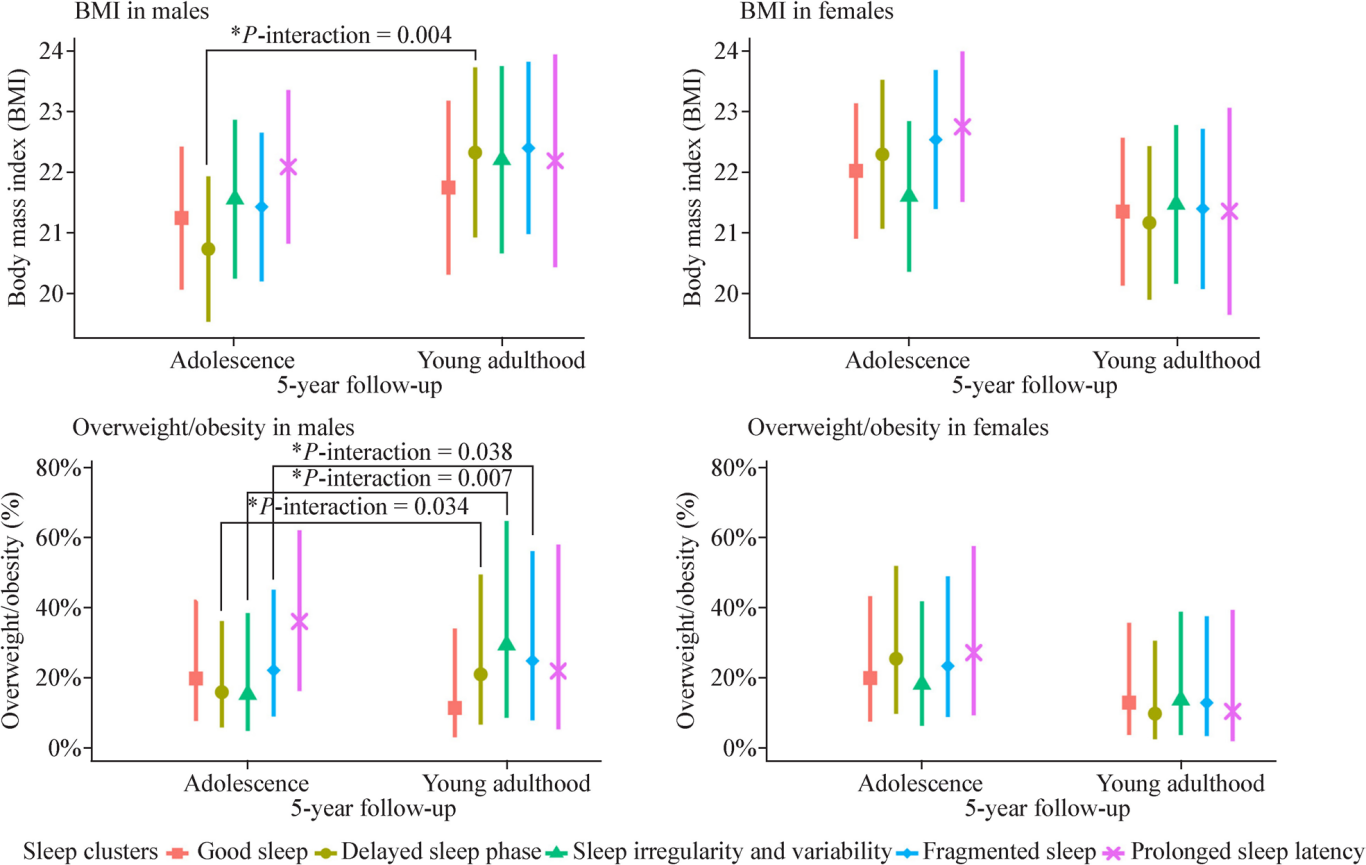
Generalized estimating equation marginal means for BMI and prevalence of overweight/obesity in males and females, with interaction terms between time of follow-ups and sleep clusters. *There is a significant interaction effect between this sleep cluster and time on the health outcomes. *BMI* body mass index

No significant interaction of time of follow-ups with sleep clusters was found in the total population and in females (Supplementary Table S8). However, among males, significant interactions were observed between time of follow-ups and the “delayed sleep phase” cluster on BMI, as well as time of follow-ups with “delayed sleep phase”, “sleep irregularity and variability” and “fragmented sleep” clusters on overweight/obesity (*P*-interaction < 0.05). Figure 3 shows the visualizations of marginal means for BMI and prevalence of overweight/obesity in five sleep clusters among males and females. These visualizations also incorporate interaction terms between sleep clusters and time of follow-ups from GEE models. In males only, marginal BMI means in the “delayed sleep phase” cluster, and overweight/obesity prevalence in “delayed sleep phase”, “sleep irregularity and variability” and “fragmented sleep” clusters display higher values in young adulthood compared to adolescence. In two cross-sectional analyses, results in adolescence aligned with GEE findings, but in young adulthood, only males exhibited associations of the “sleep irregularity and variability” and “fragmented sleep” clusters with overweight/obesity [OR = 3.84, 95% CI = (1.53, 9.64); OR = 2.62, 95% CI = (1.17, 5.85)] (Supplementary Table S9).

Main results remained largely consistent across sensitivity analyses. Results were comparable, with similar estimates but larger standard deviations due to the smaller sample sizes, when analyzing participants with repeated data (Supplementary Table S10) and excluding those with missing total energy intake (Supplementary Table S11). Supplementary Table S12 and Supplementary Table S13 confirmed the main findings when excluding participants with parent-reported BMI at 15 y and determining sleep clusters by sleep characteristics excluding weekends.

## Discussion

This study identified five consistent sleep clusters in both 1347 adolescents and 1262 young adults, including “good sleep”, “delayed sleep phase”, “sleep irregularity and variability”, “fragmented sleep”, and “prolonged sleep latency”, using K-means cluster analysis involving 12 accelerometry-assessed sleep characteristics. Adolescents within the “good sleep”, “delayed sleep phase”, and “fragmented sleep” clusters were more stable into the same cluster during the transition into young adulthood. Notably, the “prolonged sleep latency” pattern exhibited longitudinal associations with higher BMI and overweight/obesity, independent of genetic risk. Among males, the “delayed sleep phase”, “sleep irregularity and variability” and “fragmented sleep” clusters were more strongly associated with overweight/obesity as age increased.

Although TST decreased and SE increased from adolescence to young adulthood, we identified five consistent sleep patterns in both periods. Notably, we previously identified five sleep patterns in adolescence, and found a significant link between “prolonged sleep latency” cluster and higher fat mass index [14]. Now, we extend this analysis to young adults, aiming to bridge the gap in understanding objective sleep patterns during the transition from adolescence to young adulthood. This period involves significant physiological, psychological, and environmental changes, such as minimal adult supervision, independent living, more digital media use, and irregular schedule [15]. A recent study identified three sleep patterns among 2738 Brazil young adults aged 21.9–23.5 years for males (healthy sleepers, late and variant sleepers, and shorter and poorer sleepers) and for females (healthy sleepers, late and poor-quality sleepers, and shorter, variant, and inefficient sleepers), using k-means cluster analysis across seven sleep characteristics (accelerometry-measured sleep onset, offset, efficiency, TST, TST variability, the Epworth Sleep Scale and the Pittsburgh Sleep Quality Index) [13]. The “late and variant sleepers” and “late and poor-quality sleepers” clusters showed delayed sleep onset and higher SE, which was similar with our “delayed sleep phase” cluster, indicating the delayed sleep phase disorder phenotype [42]. Our current study recognized five sleep clusters in both adolescence and young adulthood, indicating consistent sleep patterns shared between these age groups. Similarly, previous studies have reported that issues like insufficient sleep, irregular sleep–wake patterns and delayed sleep phase disorder persisted from adolescence into young adulthood [16, 43]. Furthermore, the differences in subjective sleep characteristics across five sleep clusters supported our identified objective sleep clusters from a subjective perspective (Supplementary Table S3). For example, the “good sleep” cluster seemed to have higher subjective sleep quality and lower prevalence of sleep difficulties in both adolescence and young adulthood. The shorter self-reported time in bed for the “good sleep” cluster may be due to the lower SL and time awake, as the time in bed is the sum of TST, SL and time awake after sleep onset.

To our knowledge, no study has identified sleep patterns across both adolescence and young adulthood, comprehensively considering multiple objective sleep characteristics. Previous studies have either examined objective sleep patterns at one time-point [10, 11, 13], or identified subjective sleep patterns at two time-points from adolescence to young adulthood [44]. Chang et al. identified sleep categories in both adolescence and young adulthood, three for males (good sleepers, some sleep problems, poor sleepers) and two for females (good sleepers, and poor sleepers) by latent class analysis across five reported sleep problem indicators, and “good sleepers” were more stable over time [44]. Similarly, we observed that the “good sleep”, “delayed sleep phase”, and “fragmented sleep” clusters demonstrated greater stability over time. Other studies also classified consistent sleep patterns in childhood and adolescence using subjective sleep characteristics [45, 46].

The “prolonged sleep latency” cluster was longitudinally associated with higher BMI and overweight/obesity in adolescence and young adulthood, aligning with our cross-sectional findings of a link to higher adolescent fat mass [14]. A few studies examining objective sleep latency in relation to obesity had relatively small sample sizes of children and adolescents (< 600) and reported no significance [11, 47]. However, Wirth et al. reported higher BMI in individuals with sleep latency of ≥ 12 minutes (close to the median) compared to those with < 12 minutes among 430 young adults aged 21–35 years [48]. Moreover, our current research using 1347 adolescents and 1262 young adults, identified the “prolonged sleep latency” cluster, distinguished by notably higher sleep latency than other sleep clusters (mean = 45.4 vs.14.1–18.9 minutes in adolescence; 22.4 vs. 5.0–7.6 minutes in young adulthood, Table 2). As discussed in more details in our previous work [14], the association may be attributed to various mechanisms including emotional eating and more calorie intake due to anxiety, or stress from trouble falling asleep; fatigue and reduced motivation for PA caused by delayed sleep stages; hormonal imbalances like cortisol disruption affecting energy intake and expenditure [49–51].

Furthermore, among males only, relationships of the “delayed sleep phase”, “sleep irregularity and variability” and “fragmented sleep” clusters with overweight/obesity changed over time. Previous research poorly explored the evolving associations between objective sleep characteristics and obesity over time. Asarnow et al. found that reported later workday bedtime was longitudinally associated with increased BMI from adolescence to adulthood, with no age interaction [52]. However, we discovered a borderline association between the “delayed sleep phase” cluster and overweight/obesity [OR = 2.24, 95% CI = (0.98, 5.12), Supplementary Table S9] among young adult males, indicating a stronger association with BMI compared to adolescence (mean SMT = 4:24 vs. 3:24, Supplementary Table S2). Although slight changes among males from adolescence to young adulthood in “sleep irregularity and variability” and “fragmented sleep” clusters (e.g., SD in TST: 83.2 vs. 78.0 minutes; WASO/h:13.4 vs. 11.7 minutes/hour, respectively), young adult males within these clusters exhibited higher odds of having overweight/obesity. In line with an evolutionary perspective suggesting females’ higher ability to tolerate environmental stress in early life [53], our findings indicated that during the transition into young adulthood, young males may exhibit higher vulnerability to unfavorable sleep patterns and increased odds of having overweight/obesity.

In addition, PRS was independently associated with higher BMI and overweight/obesity, without sleep clusters interaction. Consistently with our prior research, PRS showed increased odds of having overweight/obesity from adolescence to young adulthood, without interaction with reported sleep duration or difficulties [39]. Similarly, in the largest study (362,496 Caucasian adults from the UK Biobank), only daytime napping, but not sleep duration or other reported sleep characteristics showed significance in interaction with PRS on BMI [54]. These results collectively suggest that independent of genetic susceptibility, nighttime sleep disturbance may be linked to increased risk of obesity.

Despite strengths like repeated measures of multiple accelerometry-assessed sleep characteristics in adolescence and young adulthood, clustering-identification of five distinct sleep clusters, availability of BMI-related genetic variants, and a large sample size, our study has several limitations. First, BMI data from a subset (*n* = 150) were parent-reported at 15 y, and were self-reported at 20 y. Including subjective BMI increased the sample size and statistical power, given a strong correlation (coefficient = 0.95) between measured and parent-reported BMI at 15 y. Olfert et al. also supported the use of self-reported anthropometric data in young adults for BMI classification [55]. Moreover, the analysis accounted for potential differences in BMI measurement methods. Second, accelerometers may tend to overestimate TST in comparison to the gold standard, polysomnography. Yet they have been validated and widely utilized as practical tools in epidemiologic studies [24, 56, 57]. Third, we assumed that one-week sleep measurements represented long-term sleep patterns, despite BMI measurements preceded sleep assessments (15 y: mean age difference = 0.38 years; 20 y: 0.28 years). Fourth, we acknowledged that some objective sleep characteristics were partly derived from subjective measures (sleep diaries). Fifth, information on daytime or evening naps, and other subjective aspects of sleep was lacking. While social jetlag and catch-up sleep were not included, we used SD in SMT and SD in TST as proxy measurements. Although we tested differences in some subjective sleep characteristics across five sleep clusters, further research could benefit from incorporating more subjective and objective sleep characteristics in a single study. Sixth, despite the longitudinal design, observational studies inherently constrain causal inference. Seventh, caution is warranted when comparing these results to others as our study involved only German adolescents aged 14–16 years and young adults aged 19–22 years. Eighth, some environmental factors, like artificial light at night, known for affecting circadian rhythms and obesity [58, 59], were unavailable for adjustment in our study. Ninth, we acknowledged that the missing of total energy intake might not be at random, despite using linear regression for imputation [35]. The sensitivity analysis excluding participants with missing data showed comparable results (Supplementary Table S11).

In conclusion, adolescents and young adults shared five consistent distinct sleep patterns, and the “prolonged sleep latency” pattern was linked to increased BMI and overweight/obesity, independent of genetic predisposition. Compared to individuals with the “good sleep” cluster, those with the “prolonged sleep latency” cluster have 1.55 times higher odds of having overweight/obesity. Young male adults with “delayed sleep phase”, “sleep irregularity and variability” and “fragmented sleep” patterns appeared to have stronger associations with overweight/obesity, compared to male adolescents. Our findings suggest that improvements on sleep latency, timing, irregularity, variability, and awakenings, may help address obesity from adolescence onward.

## Supplementary Information

Below is the link to the electronic supplementary material.

Supplementary file1 (DOCX 22599 KB)

## Data Availability

Due to data protection reasons, the datasets generated and/or analyzed during the current study cannot be made publicly available. The datasets are available to interested researchers from the corresponding author on reasonable request (e.g. reproducibility), provided the release is consistent with the consent given by the GINIplus and LISA study participants. Ethical approval might be obtained for the release and a data transfer agreement from the legal department of Helmholtz Munich must be accepted.
