# Supplementary material for: Accelerometry-assessed sleep clusters and obesity in adolescents and young adults: a longitudinal analysis in GINIplus/LISA birth cohorts
Source: World J Pediatr. 2025 Jan 4;21(1):48–61. doi: 10.1007/s12519-024-00872-5 (PMC11813820; doi:10.1007/s12519-024-00872-5)
Supplement: Supplementary file 1 — Supplementary file1 (DOCX 22599 KB) [file 12519_2024_872_MOESM1_ESM.docx]

# Online supporting information

**Accelerometry-assessed sleep clusters and obesity in adolescents and young adults: a longitudinal analysis in GINIplus/LISA birth cohorts**

**Supplementary methods**

**Method S1.** Definitions of subjective sleep characteristics.

**Supplementary results**

**Table S1**. Results of principal component analysis from the 20-year data.

**Table S2**. Sex-stratified sleep characteristics in total population and in five sleep clusters during adolescence and young adulthood.

**Table S3.** Subjective sleep characteristics in five sleep clusters during adolescence and young adulthood.

**Table S4**. Participants characteristics in each of five sleep clusters in adolescence and young adulthood.

**Table S5.** Number of participants with repeated data in sleep clusters in adolescence and young adulthood.

**Table S6.** Comparisons of identified sleep clusters by two methods in participants with repeated data in adolescence and young adulthood (*N*=636).

**Table S7.** Sex-interaction and sex-stratified associations of sleep clusters with BMI and overweight/obesity.

**Table S8.** The interactions of sleep clusters with time of follow-ups on BMI and overweight/obesity in total population and by sex.

**Table S9.** Cross-sectional associations of sleep clusters with BMI and overweight/obesity in adolescence and young adulthood, respectively.

**Table S10.** Associations of sleep clusters with BMI and overweight/obesity in participants with repeated two time-points data.

**Table S11.** Associations of sleep clusters with BMI and overweight/obesity, excluding participants with missing total energy intake information.

**Table S12.** Associations of sleep clusters with BMI and overweight/obesity, excluding participants with parent-reported BMI in adolescence.

**Table S13.** Associations of BMI and overweight/obesity with sleep clusters identified by sleep characteristics, excluding the Friday night and Saturday night.

**Fig. S1.** Hierarchical clustering dendrogram based on 12 sleep characteristics from the 20-year data.

**Fig. S2.** Sum of squares method for k-means cluster model comparison from the 20-year data.

**Fig. S3.** Principal component analysis (PCA) plots for the validation of clustering results from 15-year data on 20-year data.

## **Method S1. Definitions of subjective sleep characteristics**

Self-reported time in bed and self-reported sleep quality were assessed using the same sleep diary over seven consecutive days during accelerometry measurements in both adolescence and young adulthood. Time in bed was defined as the average total number of minutes participants spent in bed according to the diary, calculated as the difference between the in-bed and out-of-bed time, across all valid days. Sleep quality was defined as the average sleeping quality of the night (1-6; 1=worst, 6=best) according to diary, across all valid days.

Information on sleep difficulties, difficulty falling asleep, and difficulty staying asleep was collected using questionnaires filled out separately from the sleep diary. Note, the time of questionnaires completion was not at exactly the same time as the sleep diary, although both were done during the same follow-ups. In adolescence, parents reported these difficulties (yes or no), while participants provided the information in young adulthood.

| **Table S1. Results of principal component analysis** **from the 20-year data** | | | |
| --- | --- | --- | --- |
| **Component** | **Eigenvalue** | **Percentage of variance (%)** | **Cumulative percentage of variance (%)** |
| 1 | 3.99 | 33.26 | 33.26 |
| 2 | 1.89 | 15.78 | 49.04 |
| 3 | 1.50 | 12.51 | 61.55 |
| 4 | 1.13 | 9.44 | 70.99 |
| 5 | 0.94 | 7.83 | 78.82 |
| 6 | 0.80 | 6.65 | 85.47 |
| 7 | 0.72 | 6.00 | 91.47 |
| 8 | 0.60 | 4.98 | 96.45 |
| 9 | 0.20 | 1.70 | 98.15 |
| 10 | 0.19 | 1.59 | 99.74 |
| 11 | 0.03 | 0.25 | 99.99 |
| 12 | 0.00 | 0.01 | 100.00 |
| Note, the results of principal component analysis from the 15-year data can be found in previous publication [1]. | | | |

| **Table S2. Sex-stratified sleep characteristics in total population and in five sleep clusters during adolescence and young adulthood** | | | | | | | | | | | | | | | | | | |
| --- | --- | --- | --- | --- | --- | --- | --- | --- | --- | --- | --- | --- | --- | --- | --- | --- | --- | --- |
| **Sleep characteristics** | | **Total** | | **Sleep clusters** | | | | | | | | | | | | | | |
|  |  |  |  | **Good sleep** | |  | **Delayed sleep phase** | |  | **Sleep irregularity and variability** | |  | **Fragmented sleep** | |  | **Prolonged sleep latency** | | ****P*-value** |
|  |  | **Mean** | **Median [IQR]** | **Mean** | **Median [IQR]** |  | **Mean** | **Median [IQR]** |  | **Mean** | **Median [IQR]** |  | **Mean** | **Median [IQR]** |  | **Mean** | **Median [IQR]** |  |
| **Males in adolescence (14-16 years), n (%)** | | **611** | | **154 (25.2)** | |  | **101 (16.5)** | |  | **54 (8.8)** | |  | **249 (40.8)** | |  | **53 (8.7)** | |  |
|  | ***Averages across all valid days*** |  |  |  |  |  |  |  |  |  |  |  |  |  |  |  |  |  |
|  | TST, hours | 7.0 | 7.0 [0.8] | 7.4 | 7.4 [0.6] ^a^ |  | 7.1 | 7.0 [0.9] ^b^ |  | 6.7 | 6.6 [0.8] ^c^ |  | 6.8 | 6.8 [0.7] ^c^ |  | 6.7 | 6.8 [0.7] ^c^ | <0.001 |
|  | SE, % | 78.1 | 78.5 [8.9] | 84.3 | 84.2 [3.7] ^a^ |  | 82.4 | 82.5 [4.5] ^a^ |  | 73.7 | 74.3 [6.9] ^bc^ |  | 74.8 | 75.2 [5.6] ^b^ |  | 71.5 | 72.2 [7.1] ^c^ | <0.001 |
|  | SL, minutes | 18.4 | 15.4 [13.4] | 12.7 | 11.1 [9.7] ^a^ |  | 15.2 | 13.6 [9.0] ^ab^ |  | 17.2 | 15.9 [9.7] ^bc^ |  | 17.9 | 16.6 [11.2]^c^ |  | 44.1 | 41.6 [16.6] ^d^ | <0.001 |
|  | WASO/h, minutes/hour | 11.4 | 11.0 [4.8] | 8.0 | 8.1 [2.4] ^a^ |  | 9.0 | 9.0 [1.9] ^a^ |  | 14.2 | 14.0 [4.4] ^b^ |  | 13.4 | 13.1 [3.5] ^b^ |  | 13.3 | 12.7 [4.6] ^b^ | <0.001 |
|  | Awakenings/h, numbers/hour | 3.0 | 3.0 [0.8] | 2.6 | 2.6 [0.6] ^a^ |  | 2.6 | 2.7 [0.7] ^a^ |  | 2.9 | 2.9 [0.6] ^ab^ |  | 3.4 | 3.4 [0.6] ^c^ |  | 2.9 | 2.9 [0.6] ^b^ | <0.001 |
|  | SMT, 24-hour clock | 2:30 | 2:30  [54 mins] | 2:36 | 2:36  [42 mins] ^a^ |  | 3:24 | 3:24  [48 mins] ^b^ |  | 2:24 | 2:24  [42 mins] ^ac^ |  | 2:12 | 2:12  [48 mins] ^c^ |  | 2:30 | 2:36  [48 mins] ^a^ | <0.001 |
|  | ***Day-to-day variability across all valid days*** |  |  |  |  |  |  |  |  |  |  |  |  |  |  |  |  |  |
|  | SD in TST, minutes | 58.0 | 54.6 [37.0] | 48.0 | 43.8 [26.8] ^a^ |  | 81.4 | 78.3 [39.3] ^b^ |  | 83.2 | 80.7 [32.1] ^b^ |  | 47.8 | 46.6 [26.3] ^a^ |  | 65.2 | 59.9 [38.2] ^c^ | <0.001 |
|  | SD in SE, % | 5.7 | 5.2 [3.2] | 4.2 | 4.0 [1.9] ^a^ |  | 5.4 | 5.3 [2.6] ^b^ |  | 11.5 | 10.8 [4.2] ^c^ |  | 5.2 | 5.0 [2.5] ^b^ |  | 7.6 | 7.5 [3.3] ^d^ | <0.001 |
|  | SD in SL, minutes | 15.2 | 12.1 [12.8] | 9.8 | 8.3 [7.3] ^a^ |  | 12.4 | 10.3 [8.3] ^ab^ |  | 16.4 | 15.6 [15.1] ^c^ |  | 14.1 | 13.2 [10.3] ^bc^ |  | 40.3 | 36.5 [18.1] ^d^ | <0.001 |
|  | SD in WASO/h, minutes/hour | 3.4 | 3.1 [1.9] | 2.5 | 2.4 [1.2] ^a^ |  | 3.1 | 3.1 [1.5] ^b^ |  | 6.9 | 6.7 [2.2] ^c^ |  | 3.2 | 3.1 [1.7] ^b^ |  | 4.6 | 4.5 [1.7] ^d^ | <0.001 |
|  | SD in Awakenings/h, numbers/hour | 0.6 | 0.5 [0.3] | 0.5 | 0.5 [0.3] ^a^ |  | 0.7 | 0.7 [0.3] ^b^ |  | 0.7 | 0.7 [0.5] ^bc^ |  | 0.5 | 0.5 [0.2] ^a^ |  | 0.6 | 0.5 [0.3] ^ac^ | <0.001 |
|  | SD in SMT, minutes | 67.0 | 64.8 [36.1] | 54.5 | 54.0 [27.2] ^a^ |  | 99.1 | 93.5 [37.3] ^b^ |  | 76.7 | 73.0 [41.1] ^c^ |  | 59.1 | 59.8 [31.0] ^ad^ |  | 69.6 | 66.0 [35.9]^cd^ | <0.001 |
| **Females in adolescence (14-16 years), n (%)** | | **736** | | **286 (38.8)** | |  | **144 (19.6)** | |  | **76 (10.3)** | |  | **175 (23.8)** | |  | **55 (7.5)** | |  |
|  | ***Averages across all valid days*** |  |  |  |  |  |  |  |  |  |  |  |  |  |  |  |  |  |
|  | TST, hours | 7.3 | 7.3 [0.9] | 7.6 | 7.4 [0.6] ^a^ |  | 7.3 | 7.0 [0.9] ^b^ |  | 7.0 | 6.6 [0.8] ^cd^ |  | 7.0 | 6.8 [0.7] ^c^ |  | 7.2 | 6.8 [0.7] ^bd^ | <0.001 |
|  | SE, % | 80.4 | 80.7 [7.2] | 84.4 | 84.2 [3.7] ^a^ |  | 82.3 | 82.5 [4.5] ^b^ |  | 75.5 | 74.3 [6.9] ^c^ |  | 76.4 | 75.2 [5.6] ^c^ |  | 73.6 | 72.2 [7.1] ^c^ | <0.001 |
|  | SL, minutes | 18.9 | 16.0 [13.3] | 14.9 | 11.1 [9.7] ^a^ |  | 17.4 | 13.6 [9.0] ^b^ |  | 20.1 | 15.9 [9.7] ^b^ |  | 17.6 | 16.6 [11.2] ^b^ |  | 46.5 | 41.6 [16.6] ^c^ | <0.001 |
|  | WASO/h, minutes/hour | 10.0 | 9.7 [4.0] | 7.8 | 8.1 [2.4] ^a^ |  | 8.8 | 9.0 [1.9] ^b^ |  | 12.8 | 14.0 [4.4] ^c^ |  | 12.5 | 13.1 [3.5] ^c^ |  | 12.0 | 12.7 [4.6] ^c^ | <0.001 |
|  | Awakenings/h, numbers/hour | 2.7 | 2.7 [0.8] | 2.5 | 2.6 [0.6] ^a^ |  | 2.6 | 2.7 [0.7] ^ab^ |  | 2.7 | 2.9 [0.6] ^b^ |  | 3.3 | 3.4 [0.6] ^c^ |  | 2.8 | 2.9 [0.6] ^b^ | <0.001 |
|  | SMT, 24-hour clock | 2:36 | 2:30  [54 mins] | 2:30 | 2:36  [42 mins] ^a^ |  | 3:12 | 3:24  [48 mins] ^b^ |  | 2:30 | 2:24  [42 mins] ^a^ |  | 2:06 | 2:12  [48 mins] ^c^ |  | 2:24 | 2:24  [48 mins] ^a^ | <0.001 |
|  | ***Day-to-day variability across all valid days*** |  |  |  |  |  |  |  |  |  |  |  |  |  |  |  |  |  |
|  | SD in TST, minutes | 64.1 | 60.2 [37.7] | 52.6 | 43.8 [26.8] ^a^ |  | 86.6 | 78.3 [39.3] ^b^ |  | 89.5 | 80.7 [32.1] ^b^ |  | 52.1 | 46.6 [26.3] ^a^ |  | 68.5 | 59.9 [38.2] ^c^ | <0.001 |
|  | SD in SE, % | 5.5 | 4.9 [2.9] | 4.2 | 4.0 [1.9] ^a^ |  | 4.9 | 5.3 [2.6] ^b^ |  | 10.9 | 10.8 [4.2] ^c^ |  | 5.0 | 5.0 [2.5] ^b^ |  | 7.4 | 7.5 [3.3] ^d^ | <0.001 |
|  | SD in SL, minutes | 14.6 | 11.3 [11.7] | 10.6 | 8.3 [7.3] ^a^ |  | 12.5 | 10.3 [8.3] ^ab^ |  | 16.6 | 15.6 [15.1] ^c^ |  | 12.5 | 13.2 [10.3] ^bc^ |  | 45.0 | 36.5 [18.1] ^d^ | <0.001 |
|  | SD in WASO/h, minutes/hour | 3.2 | 2.9 [1.8] | 2.5 | 2.4 [1.2] ^a^ |  | 2.9 | 3.1 [1.5] ^b^ |  | 6.6 | 6.7 [2.2] ^c^ |  | 3.0 | 3.1 [1.7] ^b^ |  | 4.0 | 4.5 [1.7] ^d^ | <0.001 |
|  | SD in Awakenings/h, numbers/hour | 0.5 | 0.5 [0.2] | 0.5 | 0.5 [0.3] ^a^ |  | 0.6 | 0.7 [0.3] ^b^ |  | 0.6 | 0.7 [0.5] ^b^ |  | 0.5 | 0.5 [0.2] ^a^ |  | 0.6 | 0.5 [0.3] ^b^ | <0.001 |
|  | SD in SMT, minutes | 67.9 | 64.4 [34.9] | 57.3 | 54.0 [27.2] ^a^ |  | 94.0 | 93.5 [37.3] ^b^ |  | 73.9 | 73.0 [41.1] ^c^ |  | 59.6 | 59.8 [31.0] ^a^ |  | 73.0 | 66.0 [35.9] ^c^ | <0.001 |
| **Males in young adulthood (19-22 years), n (%)** | | **491** | | **103 (21.0)** | |  | **133 (27.1)** | |  | **60 (12.2)** | |  | **158 (32.2)** | |  | **37 (7.5)** | |  |
|  | ***Averages across all valid days*** |  |  |  |  |  |  |  |  |  |  |  |  |  |  |  |  |  |
|  | TST, hours | 6.5 | 6.4 [0.9] | 6.9 | 6.9 [0.8] ^a^ |  | 6.6 | 6.7 [0.8] ^b^ |  | 6.1 | 6.0 [0.9] ^c^ |  | 6.2 | 6.3 [0.9] ^c^ |  | 6.3 | 6.2 [1.0] ^bc^ | <0.001 |
|  | SE, % | 83.0 | 83.7 [8.3] | 88.2 | 87.7 [4.4] ^a^ |  | 87.5 | 87.7 [4.3] ^a^ |  | 78.6 | 78.8 [6.8] ^b^ |  | 79.3 | 79.7 [5.0] ^b^ |  | 75.7 | 77.8 [9.2] ^b^ | <0.001 |
|  | SL, minutes | 7.5 | 5.9 [5.9] | 5.7 | 5.3 [3.7] ^a^ |  | 5.5 | 4.6 [4.1] ^a^ |  | 8.0 | 7.7 [6.1] ^b^ |  | 6.6 | 6.0 [5.5] ^ab^ |  | 22.5 | 21.3[10.4] ^c^ | <0.001 |
|  | WASO/h, minutes/hour | 9.4 | 9.0 [4.8] | 6.4 | 6.5 [2.7] ^a^ |  | 6.9 | 6.9 [2.5] ^a^ |  | 12.0 | 11.8 [4.1] ^b^ |  | 11.7 | 11.4 [2.7] ^b^ |  | 12.5 | 11.4 [6.7] ^b^ | <0.001 |
|  | Awakenings/h, numbers/hour | 3.0 | 3.0 [1.0] | 2.5 | 2.5 [0.9] ^a^ |  | 2.5 | 2.5 [0.8] ^a^ |  | 3.1 | 3.1 [0.8] ^b^ |  | 3.6 | 3.5 [0.7] ^c^ |  | 3.3 | 3.2 [0.7] ^bc^ | <0.001 |
|  | SMT, 24-hour clock | 3:54 | 3:42  [84 mins] | 3:42 | 3:42  [84 mins] ^ab^ |  | 4:24 | 4:00  [108 mins] ^c^ |  | 4:06 | 4:00  [72 mins] ^ac^ |  | 3:30 | 3:24  [72 mins] ^b^ |  | 3:48 | 3:42  [72 mins] ^abc^ | <0.001 |
|  | ***Day-to-day variability across all valid days*** |  |  |  |  |  |  |  |  |  |  |  |  |  |  |  |  |  |
|  | SD in TST, minutes | 63.5 | 61.0 [36.8] | 46.7 | 45.0 [27.0] ^a^ |  | 78.2 | 78.2 [38.5] ^b^ |  | 78.0 | 76.6 [31.7] ^b^ |  | 55.2 | 51.3 [29.9] ^ac^ |  | 69.6 | 62.5 [35.8] ^bc^ | <0.001 |
|  | SD in SE, % | 5.2 | 4.6 [3.0] | 3.5 | 3.4 [2.0] ^a^ |  | 4.2 | 4.1 [1.9] ^b^ |  | 10.0 | 9.0 [3.2] ^c^ |  | 5.0 | 4.9 [2.1] ^d^ |  | 7.0 | 6.4 [3.6] ^e^ | <0.001 |
|  | SD in SL, minutes | 7.5 | 5.8 [5.8] | 5.3 | 4.2 [3.7] ^a^ |  | 5.4 | 4.5 [4.6] ^a^ |  | 8.8 | 8.1 [8.4] ^b^ |  | 6.3 | 6.1 [4.1] ^ab^ |  | 23.3 | 20.6 [9.0] ^c^ | <0.001 |
|  | SD in WASO/h, minutes/hour | 3.0 | 2.7 [1.8] | 2.0 | 1.9 [1.1] ^a^ |  | 2.4 | 2.3 [1.2] ^b^ |  | 5.8 | 5.4 [1.7] ^c^ |  | 3.0 | 2.9 [1.5] ^d^ |  | 3.6 | 3.3 [1.8] ^d^ | <0.001 |
|  | SD in Awakenings/h, numbers/hour | 0.7 | 0.6 [0.3] | 0.5 | 0.5 [0.3] ^a^ |  | 0.7 | 0.6 [0.4] ^b^ |  | 0.9 | 0.9 [0.5] ^c^ |  | 0.6 | 0.6 [0.3] ^b^ |  | 0.7 | 0.6 [0.3] ^b^ | <0.001 |
|  | SD in SMT, minutes | 73.4 | 68.1 [50.8] | 46.3 | 43.6 [28.1] ^a^ |  | 97.9 | 93.7 [43.5] ^b^ |  | 99.2 | 94.9 [55.5] ^b^ |  | 61.0 | 56.8 [36.7] ^c^ |  | 71.8 | 65.1 [50.5] ^c^ | <0.001 |
| **Females in young adulthood (19-22 years), n (%)** | | **771** | | **286 (37.1)** | |  | **197 (25.6)** | |  | **72 (9.3)** | |  | **182 (23.6)** | |  | **34 (4.4)** | |  |
|  | ***Averages across all valid days*** |  |  |  |  |  |  |  |  |  |  |  |  |  |  |  |  |  |
|  | TST, hours | 6.8 | 6.7 [1.0] | 7.1 | 7.1 [0.9] ^a^ |  | 6.7 | 6.7 [0.9] ^b^ |  | 6.4 | 6.4 [0.7] ^bc^ |  | 6.5 | 6.5 [0.9] ^c^ |  | 6.2 | 6.2 [0.8] ^c^ | <0.001 |
|  | SE, % | 85.2 | 85.9 [6.6] | 88.5 | 88.5 [4.0] ^a^ |  | 87.8 | 87.6 [4.3] ^a^ |  | 81.1 | 81.4 [5.1] ^b^ |  | 80.2 | 81.1 [4.5] ^b^ |  | 78.1 | 79.2 [5.7] ^b^ | <0.001 |
|  | SL, minutes | 6.3 | 5.0 [5.6] | 4.7 | 3.8 [3.9] ^a^ |  | 5.3 | 4.4 [4.5] ^a^ |  | 7.4 | 7.4 [6.2] ^b^ |  | 6.6 | 6.1 [5.6] ^b^ |  | 22.3 | 20.8 [6.4] ^c^ | <0.001 |
|  | WASO/h, minutes/hour | 8.2 | 7.8 [3.8] | 6.4 | 6.4 [2.5] ^a^ |  | 6.7 | 6.8 [2.4] ^a^ |  | 10.5 | 10.5 [3.6] ^b^ |  | 11.2 | 10.8 [2.7] ^b^ |  | 10.7 | 9.8 [4.2] ^b^ | <0.001 |
|  | Awakenings/h, numbers/hour | 2.8 | 2.8 [0.9] | 2.5 | 2.6 [0.7] ^a^ |  | 2.5 | 2.5 [0.7] ^a^ |  | 2.8 | 2.8 [0.7] ^b^ |  | 3.5 | 3.5 [0.6] ^c^ |  | 2.9 | 2.9 [0.7] ^b^ | <0.001 |
|  | SMT, 24-hour clock | 3:24 | 3:18  [78 mins] | 3:18 | 3:12  [78 mins] ^a^ |  | 4:00 | 4:00  [78 mins] ^b^ |  | 3:24 | 3:18  [78 mins] ^a^ |  | 3:06 | 3:06  [60 mins] ^a^ |  | 3:36 | 3:24  [66 mins] ^ab^ | <0.001 |
|  | ***Day-to-day variability across all valid days*** |  |  |  |  |  |  |  |  |  |  |  |  |  |  |  |  |  |
|  | SD in TST, minutes | 65.6 | 61.1 [36.8] | 51.5 | 49.3 [26.9] ^a^ |  | 83.8 | 82.2 [32.1] ^b^ |  | 89.6 | 88.7 [48.4] ^b^ |  | 57.2 | 54.9 [27.1] ^ac^ |  | 72.3 | 62 [27.2] ^bc^ | <0.001 |
|  | SD in SE, % | 4.7 | 4.2 [2.6] | 3.3 | 3.1 [1.6] ^a^ |  | 4.3 | 4.1 [1.9] ^b^ |  | 9.8 | 9.0 [2.6] ^c^ |  | 5.0 | 5.0 [1.8] ^d^ |  | 6.4 | 5.6 [3.1] ^d^ | <0.001 |
|  | SD in SL, minutes | 6.3 | 4.9 [4.7] | 4.5 | 3.8 [3.2] ^a^ |  | 5.3 | 4.7 [4.1] ^ab^ |  | 8.9 | 8.3 [7.9] ^c^ |  | 6.2 | 5.6 [4.3] ^bc^ |  | 22.2 | 19.2 [4.8] ^d^ | <0.001 |
|  | SD in WASO/h, minutes/hour | 2.8 | 2.5 [1.4] | 2.0 | 1.9 [0.9] ^a^ |  | 2.5 | 2.4 [1.1] ^b^ |  | 5.8 | 5.1 [1.8] ^c^ |  | 3.0 | 3.0 [1.1] ^d^ |  | 3.1 | 3.0 [1.3] ^bd^ | <0.001 |
|  | SD in Awakenings/h, numbers/hour | 0.6 | 0.6 [0.3] | 0.5 | 0.5 [0.2] ^a^ |  | 0.7 | 0.6 [0.3] ^b^ |  | 0.8 | 0.8 [0.4] ^c^ |  | 0.6 | 0.6 [0.3] ^b^ |  | 0.6 | 0.6 [0.2] ^ab^ | <0.001 |
|  | SD in SMT, minutes | 68.5 | 63.6 [42.4] | 51.2 | 48.9 [29.9] ^a^ |  | 92.3 | 87.7 [41.1] ^b^ |  | 87.8 | 85.2 [47] ^bc^ |  | 62.8 | 58.5 [38.3] ^d^ |  | 66.9 | 64.5 [41.5]^cd^ | <0.001 |
| The results are presented as mean and median [IQR] of sleep characteristics, given that most of them did not follow a normal distribution. *: Kruskal-Wallis test with Dunn post hoc tests and Bonferroni adjustment. Sharing the same letter (a b c d e) are considered not significantly different (adjusted *P*-values < 0.05). Abbreviations: Awakenings/h, awakenings per hour after sleep onset; IQR, interquartile range; SD, standard deviation; SE, sleep efficiency; SL, sleep latency; SMT, sleep midpoint timing; TST, total sleep time; WASO/h, time awake per hour after sleep onset. | | | | | | | | | | | | | | | | | | |

| **Table S3.** **Subjective sleep characteristics in five sleep clusters during adolescence and young adulthood** | | | | | | | | |
| --- | --- | --- | --- | --- | --- | --- | --- | --- |
| **Sleep characteristics** | | **Sleep clusters** | | | | | |  |
|  |  | **Good sleep** | **Delayed sleep phase** | **Sleep irregularity and variability** | **Fragmented sleep** | **Prolonged sleep latency** | ****P*-value** | **Number of participants** |
| **Adolescence (14-16 years)** | |  |  |  |  |  |  |  |
|  | Self-reported time in bed, hours | 8.95 ± 0.70 ^a^ | 8.80 ± 0.86 ^a^ | 9.24 ± 0.84 ^b^ | 9.15 ± 0.77 ^b^ | 9.68 ± 1.00 ^c^ | **<0.001** | 1347 |
|  | Self-reported sleep quality | 4.67 ± 0.72 ^a^ | 4.56 ± 0.68 ^ab^ | 4.46 ± 0.75 ^b^ | 4.59 ± 0.67 ^ab^ | 4.46 ± 0.66 ^b^ | **0.004** | 1347 |
|  | Parent-reported sleep difficulties, yes | 57 (13.2) | 25 (10.4) | 23 (18.9) | 55 (13.2) | 18 (17.3) | 0.180 | 1313 |
|  | Parent-reported difficulty falling asleep, yes | 52 (12.1) | 24 (10.0) | 19 (15.8) | 49 (11.8) | 17 (16.3) | 0.370 | 1311 |
|  | Parent-reported difficulty staying asleep, yes | 16 (3.7) | 4 (1.7) | 5 (4.2) | 10 (2.4) | 2 (1.9) | 0.437 | 1311 |
| **Young adulthood (19-22 years)** | |  |  |  |  |  |  |  |
|  | Self-reported time in bed, hours | 8.01 ± 0.76 ^a^ | 7.61 ± 0.75 ^b^ | 7.85 ± 0.95 ^a^ | 7.97 ± 0.80 ^a^ | 8.17 ± 0.90 ^a^ | **<0.001** | 1262 |
|  | Self-reported sleep quality | 4.43 ± 0.82 | 4.35 ± 0.83 | 4.26 ± 0.83 | 4.33 ± 0.75 | 4.21 ± 0.83 | 0.137 | 1262 |
|  | Self-reported sleep difficulties, yes | 74 (19.8) | 68 (21.8) | 36 (29.8) | 63 (19.6) | 19 (28.4) | 0.095 | 1194 |
|  | Self-reported difficulty falling asleep, yes | 53 (14.2) | 58 (18.6) | 29 (24.0) | 45 (14.0) | 16 (23.9) | **0.025** | 1193 |
|  | Self-reported difficulty staying asleep, yes | 35 (9.4) | 29 (9.3) | 14 (11.6) | 35 (10.9) | 9 (13.4) | 0.788 | 1193 |
| The results are presented as mean ± SD or number (%) of sleep characteristics. *: Post hoc tests after one-way analysis of variance or Chi-square test, with Bonferroni adjustment. Sharing the same letter (a b c) are considered not significantly different (adjusted *P*-values < 0.05). *P*-values < 0.05 were highlighted in bold. Self-reported sleep quality was presented on a scale of 1 (worst) to 6 (best) according to the sleep diary. Self-reported time in bed and sleep quality represented averages assessed from the sleep diary across valid days. Sleep difficulties, difficulty falling asleep, and difficulty staying asleep were assessed by questionnaires. | | | | | | | | |

| **Table S4. Participants characteristics in each of five sleep clusters in adolescence and young adulthood** | | | | | | |
| --- | --- | --- | --- | --- | --- | --- |
|  | **Good sleep** | **Delayed sleep phase** | **Sleep irregularity and variability** | **Fragmented sleep** | **Prolonged sleep latency** | ****P*-value** |
| **Adolescence** | 440 (32.7) | 245 (18.2) | 130 (9.6) | 424 (31.5) | 108 (8.0) |  |
| Age, year | 15.2 ± 0.3 | 15.2 ± 0.3 | 15.2 ± 0.3 | 15.2 ± 0.3 | 15.2 ± 0.3 | 0.538 |
| Study, n(%) |  |  |  |  |  | **0.018** |
| GINIplus observation | 173 (39.3) | 103 (42.0) | 42 (32.3) | 140 (33.0) | 39 (36.1) |  |
| GINIplus intervention | 171 (38.9) | 93 (38.0) | 53 (40.8) | 149 (35.1) | 43 (39.8) |  |
| LISA | 96 (21.8) | 49 (20.0) | 35 (26.9) | 135 (31.8) | 26 (24.1) |  |
| Study center, n(%) |  |  |  |  |  | **0.003** |
| Munich | 278 (63.2) | 130 (53.1) | 70 (53.8) | 280 (66.0) | 60 (55.6) |  |
| Wesel | 162 (36.8) ^ab^ | 115 (46.9) ^a^ | 60 (46.2) ^ab^ | 144 (34.0) ^b^ | 48 (44.4) ^ab^ |  |
| Weight, kg | 59.9 ± 10.8 ^a^ | 60.7 ± 10.8 ^ab^ | 61.8 ± 11.5 ^ab^ | 61.8 ± 10.7 ^ab^ | 63.2 ± 13.0 ^b^ | **0.019** |
| Height, cm | 170.4 ± 8.3 ^a^ | 171.5 ± 7.9 ^ab^ | 171.3 ± 7.8 ^ab^ | 172.4 ± 7.8 ^b^ | 171.5 ± 8.7 ^ab^ | **0.007** |
| BMI, kg/m^2^ | 20.6 ± 2.9 | 20.6 ± 3.1 | 21.0 ± 3.2 | 20.7 ± 3.0 | 21.4 ± 3.4 | 0.089 |
| BMI z-score | 0.0 ± 1.0 | 0.0 ± 1.0 | 0.1 ± 0.9 | 0.1 ± 1.0 | 0.3 ± 1.0 | 0.056 |
| Overweight/obesity, n(%) |  |  |  |  |  | 0.169 |
| No | 374 (85.0) | 204 (83.3) | 110 (84.6) | 351 (82.8) | 81 (75.0) |  |
| Yes | 66 (15.0) | 41 (16.7) | 20 (15.4) | 73 (17.2) | 27 (25.0) |  |
| BMI measurement methods, n(%) |  |  |  |  |  | 0.191 |
| Examination | 397 (90.2) | 219 (89.4) | 119 (91.5) | 364 (85.8) | 98 (90.7) |  |
| Questionnaire | 43 (9.8) | 26 (10.6) | 11 (8.5) | 60 (14.2) | 10 (9.3) |  |
| Season, n(%) |  |  |  |  |  | **0.032** |
| Spring | 110 (25.0) | 78 (31.8) | 34 (26.2) | 109 (25.7) | 24 (22.2) |  |
| Summer | 50 (11.4) | 33 (13.5) | 24 (18.5) | 69 (16.3) | 22 (20.4) |  |
| Autumn | 146 (33.2) | 72 (29.4) | 39 (30.0) | 152 (35.8) | 28 (25.9) |  |
| Winter | 134 (30.5) | 62 (25.3) | 33 (25.4) | 94 (22.2) | 34 (31.5) |  |
| Total energy intake, kcal/day | 1998.7 ± 628.7 ^a^ | 2020.4 ± 625.8 ^ab^ | 2129.4 ± 663.2 ^ab^ | 2173.5 ± 673.3 ^b^ | 2088.9 ± 610.6 ^ab^ | **0.005** |
| Sedentary behavior, hours | 8.5 ± 1.3 ^ab^ | 8.6 ± 1.5 ^a^ | 8.2 ± 1.2 ^bc^ | 8.0 ± 1.4 ^c^ | 7.5 ± 1.3 ^d^ | **<0.001** |
| MVPA, mins | 48.3 ± 26.1 ^a^ | 49.8 ± 34.3 ^ab^ | 49.3 ± 23.6 ^ab^ | 54.3 ± 25.0 ^b^ | 52.2 ± 23.5 ^ab^ | **0.019** |
| Parental highest education, n(%) |  |  |  |  |  | **0.001** |
| Low/medium | 111 (25.2) | 72 (29.4) | 47 (36.2) | 117 (27.6) | 48 (44.4) |  |
| High | 329 (74.8) ^ab^ | 173 (70.6) ^ab^ | 83 (63.8) ^ab^ | 307 (72.4) ^a^ | 60 (55.6) ^b^ |  |
| **Young adulthood** | 389 (30.8) | 330 (26.2) | 132 (10.5) | 340 (26.9) | 71 (5.6) |  |
| Age, year | 20.2 ± 0.4 ^a^ | 20.2 ± 0.4 ^ab^ | 20.3 ± 0.5 ^b^ | 20.2 ± 0.4 ^ab^ | 20.2 ± 0.3 ^ab^ | **0.003** |
| Study, n(%) |  |  |  |  |  | 0.419 |
| GINIplus observation | 166 (42.7) | 121 (36.7) | 55 (41.7) | 120 (35.3) | 26 (36.6) |  |
| GINIplus intervention | 136 (35.0) | 139 (42.1) | 46 (34.8) | 131 (38.5) | 28 (39.4) |  |
| LISA | 87 (22.4) | 70 (21.2) | 31 (23.5) | 89 (26.2) | 17 (23.9) |  |
| Study center, n(%) |  |  |  |  |  | 0.114 |
| Munich | 244 (62.7) | 201 (60.9) | 66 (50.0) | 205 (60.3) | 46 (64.8) |  |
| Wesel | 145 (37.3) | 129 (39.1) | 66 (50.0) | 135 (39.7) | 25 (35.2) |  |
| Weight, kg | 66.5 ± 11.1 ^a^ | 68.7 ± 13.2 ^ab^ | 72.0 ± 13.8 ^b^ | 69.9 ± 12.6 ^b^ | 70.0 ± 13.9 ^ab^ | **<0.001** |
| Height, cm | 172.9 ± 9.3 ^a^ | 174.2 ± 9.3 ^ab^ | 176.9 ± 10.2 ^b^ | 175.6 ± 9.4 ^b^ | 175.6 ± 10.7 ^ab^ | **<0.001** |
| BMI, kg/m^2^ | 22.2 ± 3.0 | 22.6 ± 3.5 | 22.9 ± 3.5 | 22.6 ± 3.4 | 22.6 ± 3.4 | 0.162 |
| Overweight/obesity, n(%) |  |  |  |  |  | 0.349 |
| No | 326 (83.8) | 277 (83.9) | 101 (76.5) | 277 (81.5) | 59 (83.1) |  |
| Yes | 63 (16.2) | 53 (16.1) | 31 (23.5) | 63 (18.5) | 12 (16.9) |  |
| BMI measurements, n(%) |  |  |  |  |  | NA |
| Examination |  |  |  |  |  |  |
| Questionnaire | 389 (100.0) | 330 (100.0) | 132 (100.0) | 340 (100.0) | 71 (100.0) |  |
| Season, n(%) |  |  |  |  |  | **0.047** |
| Spring | 110 (28.3) | 91 (27.6) | 31 (23.5) | 99 (29.1) | 18 (25.4) |  |
| Summer | 88 (22.6) | 83 (25.2) | 47 (35.6) | 105 (30.9) | 19 (26.8) |  |
| Autumn | 86 (22.1) | 83 (25.2) | 29 (22.0) | 81 (23.8) | 18 (25.4) |  |
| Winter | 105 (27.0) | 73 (22.1) | 25 (18.9) | 55 (16.2) | 16 (22.5) |  |
| Total energy intake, kcal/day | 1720.3 ± 611.5 | 1799.2 ± 678.5 | 1776.0 ± 622.6 | 1801.6 ± 725.2 | 1887.0 ± 595.5 | 0.331 |
| Sedentary behavior, hours | 8.7 ± 1.5 ^a^ | 8.5 ± 1.6 ^ab^ | 8.1 ± 1.5 ^bc^ | 8.2 ± 1.5 ^bc^ | 7.8 ± 1.5 ^c^ | **<0.001** |
| MVPA, minutes | 45.3 ± 24.2 | 47.9 ± 23.0 | 44.8 ± 23.4 | 46.5 ± 24.5 | 45.4 ± 20.1 | 0.565 |
| Parental highest education, n(%) |  |  |  |  |  | **0.015** |
| Low/medium | 88 (22.6) | 103 (31.2) | 47 (35.6) | 93 (27.4) | 24 (33.8) |  |
| High | 301 (77.4) ^a^ | 227 (68.8) ^ab^ | 85 (64.4) ^b^ | 247 (72.6) ^ab^ | 47 (66.2) ^ab^ |  |
| The results are presented as mean ± standard deviation or n (%) (number(percentage)). Abbreviations: BMI, body mass index; MVPA, moderate-to-vigorous physical activity; GINIplus, German Infant Study on the influence of Nutrition Intervention PLUS environmental and genetic influences on allergy development; LISA, Influence of Lifestyle factors on the development of the Immune System and Allergies in East and West Germany.  Overweight/obesity: BMI z-score >1 for adolescents; BMI ≥25 kg/m^2^ for adults according to World Health Organization. | | | | | | |
| The number of participants with available total energy intake: n=1063 in adolescence; n=1019 in young adulthood. | | | | | | |
| *: Post hoc tests after one-way analysis of variance or Chi-square test, with Bonferroni adjustment. Sharing the same letter (a b c) are considered not significantly different (adjusted *P*-values < 0.05). *P*-values < 0.05 were highlighted in bold. | | | | | | |

| **Table S5. Number of participants with repeated data in sleep clusters in adolescence and young adulthood** | | | | | |
| --- | --- | --- | --- | --- | --- |
| **Sleep clusters in adolescence** | **Sleep clusters in young adulthood** | | | | |
|  | Good sleep | Delayed sleep phase | Sleep irregularity and variability | Fragmented sleep | Prolonged sleep latency |
| **Total (n=636)** |  |  |  |  |  |
| Good sleep | 119 (0.50) | 73 (0.31) | 16 (0.07) | 27 (0.11) | 4 (0.02) |
| Delayed sleep phase | 33 (0.31) | 42 (0.40) | 6 (0.06) | 21 (0.20) | 4 (0.04) |
| Sleep irregularity and variability | 11 (0.21) | 16 (0.30) | 5 (0.09) | 16 (0.30) | 5 (0.09) |
| Fragmented sleep | 31 (0.16) | 27 (0.14) | 30 (0.15) | 94 (0.48) | 12 (0.06) |
| Prolonged sleep latency | 8 (0.18) | 12 (0.27) | 7 (0.16) | 15 (0.34) | 2 (0.05) |
| **Male (n=243)** |  |  |  |  |  |
| Good sleep | 32 (0.42) | 24 (0.32) | 6 (0.08) | 14 (0.18) | 0 (0) |
| Delayed sleep phase | 5 (0.16) | 15 (0.47) | 2 (0.06) | 9 (0.28) | 1 (0.03) |
| Sleep irregularity and variability | 0 (0) | 7 (0.44) | 2 (0.13) | 5 (0.31) | 2 (0.13) |
| Fragmented sleep | 13 (0.13) | 18 (0.18) | 16 (0.16) | 43 (0.43) | 9 (0.09) |
| Prolonged sleep latency | 5 (0.25) | 6 (0.30) | 1 (0.05) | 7 (0.35) | 1 (0.05) |
| **Female (n=393)** |  |  |  |  |  |
| Good sleep | 87 (0.53) | 49 (0.30) | 10 (0.06) | 13 (0.08) | 4 (0.02) |
| Delayed sleep phase | 28 (0.38) | 27 (0.36) | 4 (0.05) | 12 (0.16) | 3 (0.04) |
| Sleep irregularity and variability | 11 (0.30) | 9 (0.24) | 3 (0.08) | 11 (0.30) | 3 (0.08) |
| Fragmented sleep | 18 (0.19) | 9 (0.09) | 14 (0.15) | 51 (0.54) | 3 (0.03) |
| Prolonged sleep latency | 3 (0.13) | 6 (0.25) | 6 (0.25) | 8 (0.33) | 1 (0.04) |
| The results are presented as number (proportion) within the group of sleep clusters in adolescence. | | | | | |

| **Table S6. Comparisons of identified sleep clusters by two methods in participants with repeated data in adolescence and young adulthood (*N*=636)** | | | | | |
| --- | --- | --- | --- | --- | --- |
| **Sleep clusters identified at each follow-up using all available data at that time** | **Sleep clusters re-identified only among participants with repeated data (N=636)** | | | | |
|  | Good sleep | Delayed sleep phase | Sleep irregularity and variability | Fragmented sleep | Prolonged sleep latency |
| **Sleep clusters in adolescence** |  |  |  |  |  |
| Good sleep | **203** | 28 | 2 | 6 | 0 |
| Delayed sleep phase | 1 | **104** | 1 | 0 | 0 |
| Sleep irregularity and variability | 0 | 0 | **49** | 2 | 2 |
| Fragmented sleep | 0 | 10 | 0 | **184** | 0 |
| Prolonged sleep latency | 1 | 2 | 1 | 3 | **37** |
| **Sleep clusters in young adulthood** |  |  |  |  |  |
| Good sleep | **187** | 3 | 0 | 12 | 0 |
| Delayed sleep phase | 53 | **116** | 0 | 1 | 0 |
| Sleep irregularity and variability | 0 | 15 | **45** | 4 | 0 |
| Fragmented sleep | 0 | 11 | 0 | **161** | 1 |
| Prolonged sleep latency | 0 | 2 | 0 | 0 | **25** |
| The results are presented as number of participants within the group of sleep clusters identified by two methods. The number of participants remained in the same cluster were highlighted in bold. The Cohen’s kappa of clustering results identified by the two methods was 0.87 in adolescence and 0.78 in young adulthood, both statistically significant (*P*-values < 0.001). | | | | | |

| **Table S7. Sex-interaction and sex-stratified associations of sleep clusters with BMI and overweight/obesity** | | | | | | | |  |  |
| --- | --- | --- | --- | --- | --- | --- | --- | --- | --- |
| **Sleep clusters** | **Male** | | |  | **Female** | | |  | **Sex-interaction** |
| **BMI** | **Observations** | **β [95%CI]** | ***P*-value** |  | **Observations** | **β [95%CI]** | ***P*-value** |  | ***P*-value** |
| Good sleep | 257 | Ref |  |  | 572 | Ref |  |  |  |
| Delayed sleep phase | 234 | 0.08 [-0.31, 0.47] | 0.693 |  | 341 | 0.04 [-0.30, 0.37] | 0.837 |  | 0.325 |
| Sleep irregularity and variability | 114 | 0.34 [-0.18, 0.86] | 0.204 |  | 148 | -0.15 [-0.58, 0.28] | 0.482 |  | 0.146 |
| Fragmented sleep | 407 | 0.39 [-0.00, 0.78] | 0.051 |  | 357 | 0.26 [-0.12, 0.64] | 0.180 |  | 0.652 |
| Prolonged sleep latency | 90 | 0.69 [-0.07, 1.45] | 0.075 |  | 89 | 0.43 [-0.21, 1.07] | 0.191 |  | 0.719 |
| **Overweight/obesity** | **Cases**  **/Observations** | **OR [95%CI]** | ***P*-value** |  | **Cases**  **/Observations** | **OR [95%CI]** | ***P*-value** |  | ***P*-value** |
| Good sleep | 14.8% | Ref |  |  | 15.9% | Ref |  |  |  |
| Delayed sleep phase | 17.9% | 1.15 [0.74, 1.81] | 0.530 |  | 15.2% | 0.96 [0.70, 1.32] | 0.801 |  | 0.655 |
| Sleep irregularity and variability | 21.9% | 1.48 [0.87, 2.52] | 0.151 |  | 17.6% | 0.97 [0.62, 1.52] | 0.894 |  | 0.324 |
| Fragmented sleep | 20.4% | 1.51 [1.01, 2.26] | **0.046** |  | 14.8% | 1.08 [0.75, 1.56] | 0.686 |  | 0.183 |
| Prolonged sleep latency | 27.8% | 2.20 [1.26, 3.84] | **0.005** |  | 15.7% | 1.12 [0.56, 2.23] | 0.757 |  | 0.088 |
| Adjusted for time of follow-ups, age, study, study center, parental highest education, BMI measurement methods, season, sedentary behavior, moderate-to-vigorous physical activity, and total energy intake. *P*-values < 0.05 were highlighted in bold. Abbreviations: BMI, body mass index; CI, confidence interval; OR, odds ratio. | | | | | | | | | |

| **Table S8. The interactions of sleep clusters with time of follow-ups on BMI and overweight/obesity in total population and by sex** | | | | | | | | |
| --- | --- | --- | --- | --- | --- | --- | --- | --- |
| **Sleep clusters** | **Total** | |  | **Male** | |  | **Female** | |
| **BMI** | **β [95%CI]** | ***P*-value** |  | **β [95%CI]** | ***P*-value** |  | **β [95%CI]** | ***P*-value** |
| Good sleep | Ref |  |  | Ref |  |  | Ref |  |
| Delayed sleep phase | -0.04 [-0.42, 0.34] | 0.837 |  | -0.51 [-1.08, 0.06] | 0.080 |  | 0.27 [-0.22, 0.76] | 0.273 |
| Sleep irregularity and variability | -0.10 [-0.55, 0.35] | 0.657 |  | 0.31 [-0.39, 1.01] | 0.384 |  | -0.42 [-1.06, 0.23] | 0.204 |
| Fragmented sleep | 0.19 [-0.12, 0.51] | 0.225 |  | 0.18 [-0.28, 0.65] | 0.443 |  | 0.52 [0.10, 0.94] | **0.015** |
| Prolonged sleep latency | 0.68 [0.16, 1.21] | **0.011** |  | 0.84 [-0.03, 1.72] | 0.060 |  | 0.73 [0.11, 1.35] | **0.021** |
| Time of follow-ups | -0.45 [-1.94, 1.04] | 0.556 |  | 0.50 [-1.68, 2.68] | 0.654 |  | -0.67 [-2.61, 1.27] | 0.499 |
| Delayed sleep phase * Time of follow-ups | 0.21 [-0.31, 0.73] | 0.425 |  | 1.09 [0.35, 1.83] | **0.004** |  | -0.46 [-1.13, 0.22] | 0.184 |
| Sleep irregularity and variability * Time of follow-ups | 0.41 [-0.25, 1.06] | 0.226 |  | 0.15 [-0.79, 1.08] | 0.757 |  | 0.54 [-0.40, 1.48] | 0.261 |
| Fragmented sleep * Time of follow-ups | 0.16 [-0.26, 0.59] | 0.454 |  | 0.47 [-0.14, 1.09] | 0.134 |  | -0.47 [-1.01, 0.07] | 0.088 |
| Prolonged sleep latency * Time of follow-ups | -0.34 [-1.39, 0.70] | 0.519 |  | -0.40 [-1.92, 1.12] | 0.605 |  | -0.72 [-2.16, 0.71] | 0.324 |
| **Overweight/obesity** | **OR [95%CI]** | ***P*-value** |  | **OR [95%CI]** | ***P*-value** |  | **OR [95%CI]** | ***P*-value** |
| Good sleep | Ref |  |  | Ref |  |  | Ref |  |
| Delayed sleep phase | 1.05 [0.71, 1.55] | 0.811 |  | 0.77 [0.40, 1.47] | 0.423 |  | 1.37 [0.84, 2.23] | 0.208 |
| Sleep irregularity and variability | 0.83 [0.49, 1.42] | 0.503 |  | 0.73 [0.33, 1.63] | 0.442 |  | 0.89 [0.44, 1.81] | 0.750 |
| Fragmented sleep | 1.20 [0.86, 1.68] | 0.279 |  | 1.15 [0.72, 1.85] | 0.557 |  | 1.22 [0.74, 2.01] | 0.428 |
| Prolonged sleep latency | 1.95 [1.20, 3.18] | **0.007** |  | 2.29 [1.20, 4.34] | **0.012** |  | 1.50 [0.67, 3.37] | 0.329 |
| Time of follow-ups | 0.65 [0.16, 2.73] | 0.557 |  | 0.52 [0.06, 4.27] | 0.544 |  | 0.60 [0.08, 4.59] | 0.620 |
| Delayed sleep phase * Time of follow-ups | 0.92 [0.54, 1.57] | 0.758 |  | 2.69 [1.08, 6.73] | **0.034** |  | 0.53 [0.27, 1.04] | 0.066 |
| Sleep irregularity and variability * Time of follow-ups | 1.75 [0.89, 3.45] | 0.106 |  | 4.43 [1.51, 13.02] | **0.007** |  | 1.19 [0.48, 2.96] | 0.701 |
| Fragmented sleep * Time of follow-ups | 1.03 [0.65, 1.63] | 0.915 |  | 2.23 [1.05, 4.74] | **0.037** |  | 0.81 [0.43, 1.52] | 0.519 |
| Prolonged sleep latency * Time of follow-ups | 0.54 [0.23, 1.28] | 0.162 |  | 0.96 [0.30, 3.05] | 0.938 |  | 0.52 [0.13, 2.07] | 0.357 |
| Adjusted for sex (only for total population), age, study, study center, parental highest education, BMI measurement methods, season, sedentary behavior, moderate-to-vigorous physical activity, and total energy intake. *P*-values < 0.05 were highlighted in bold. Abbreviations: BMI, body mass index; CI, confidence interval; OR, odds ratio. | | | | | | | | |

| **Table S9. Cross-sectional associations of sleep clusters with BMI and overweight/obesity in adolescence and young adulthood, respectively** | | | | | | | | | | | |
| --- | --- | --- | --- | --- | --- | --- | --- | --- | --- | --- | --- |
| **Sleep clusters** | **Total** | | |  | **Male** | | |  | **Female** | | |
| ***Adolescence*** |  |  |  |  |  |  |  |  |  |  |  |
| **BMI** | **Numbers** | **β [95%CI]** | ***P*** |  | **Numbers** | **β [95%CI]** | ***P*** |  | **Numbers** | **β [95%CI]** | ***P*** |
| Good sleep | 440 | Ref |  |  | 154 | Ref |  |  | 286 | Ref |  |
| Delayed sleep phase | 245 | -0.12 [-0.58, 0.34] | 0.614 |  | 101 | -0.47 [-1.24, 0.30] | 0.228 |  | 144 | 0.10 [-0.49, 0.69] | 0.738 |
| Sleep irregularity and variability | 130 | 0.44 [-0.15, 1.02] | 0.141 |  | 54 | 0.37 [-0.58, 1.33] | 0.442 |  | 76 | 0.46 [-0.28, 1.20] | 0.226 |
| Fragmented sleep | 424 | 0.41 [0.00, 0.81] | 0.050 |  | 249 | 0.30 [-0.33, 0.92] | 0.349 |  | 175 | 0.45 [-0.11, 1.00] | 0.114 |
| Prolonged sleep latency | 108 | 1.03 [0.39, 1.67] | **0.002** |  | 53 | 1.13 [0.15, 2.12] | **0.024** |  | 55 | 0.87 [0.01, 1.73] | **0.047** |
| **Overweight/obesity** | **Prevalence** | **OR [95%CI]** | ***P*** |  | **Prevalence** | **OR [95%CI]** | ***P*** |  | **Prevalence** | **OR [95%CI]** | ***P*** |
| Good sleep | 15.0% | Ref |  |  | 18.8% | Ref |  |  | 12.9% | Ref |  |
| Delayed sleep phase | 16.7% | 0.98 [0.63, 1.52] | 0.931 |  | 16.8% | 0.76 [0.39, 1.51] | 0.437 |  | 16.7% | 1.21 [0.68, 2.14] | 0.517 |
| Sleep irregularity and variability | 15.4% | 0.98 [0.56, 1.72] | 0.956 |  | 14.8% | 0.69 [0.29, 1.67] | 0.413 |  | 15.8% | 1.27 [0.61, 2.64] | 0.517 |
| Fragmented sleep | 17.2% | 1.18 [0.80, 1.74] | 0.394 |  | 20.5% | 1.14 [0.67, 1.95] | 0.622 |  | 12.6% | 1.17 [0.65, 2.09] | 0.608 |
| Prolonged sleep latency | 25.0% | 1.99 [1.16, 3.41] | **0.013** |  | 34.0% | 2.21 [1.04, 4.70] | **0.040** |  | 16.4% | 1.58 [0.69, 3.65] | 0.279 |
| ***Young adulthood*** |  |  |  |  |  |  |  |  |  |  |  |
| **BMI** | **Numbers** | **β [95%CI]** | ***P*** |  | **Numbers** | **β [95%CI]** | ***P*** |  | **Numbers** | **β [95%CI]** | ***P*** |
| Good sleep | 389 | Ref |  |  | 103 | Ref |  |  | 286 | Ref |  |
| Delayed sleep phase | 330 | 0.22 [-0.26, 0.70] | 0.373 |  | 133 | 0.75 [-0.00, 1.50] | 0.052 |  | 197 | -0.00 [-0.63, 0.62] | 0.990 |
| Sleep irregularity and variability | 132 | 0.51 [-0.15, 1.17] | 0.128 |  | 60 | 0.83 [-0.12, 1.78] | 0.088 |  | 72 | 0.50 [-0.40, 1.40] | 0.274 |
| Fragmented sleep | 340 | 0.33 [-0.16, 0.82] | 0.184 |  | 158 | 0.71 [-0.02, 1.44] | 0.059 |  | 182 | 0.10 [-0.55, 0.75] | 0.764 |
| Prolonged sleep latency | 71 | 0.31 [-0.53, 1.14] | 0.473 |  | 37 | 0.47 [-0.64, 1.58] | 0.408 |  | 34 | 0.20 [-1.02, 1.43] | 0.745 |
| **Overweight/obesity** | **Prevalence** | **OR [95%CI]** | ***P*** |  | **Prevalence** | **OR [95%CI]** | ***P*** |  | **Prevalence** | **OR [95%CI]** | ***P*** |
| Good sleep | 16.2% | Ref |  |  | 8.7% | Ref |  |  | 18.9% | Ref |  |
| Delayed sleep phase | 16.1% | 0.93 [0.62, 1.40] | 0.736 |  | 18.8% | 2.24 [0.98, 5.12] | 0.055 |  | 14.2% | 0.69 [0.42, 1.15] | 0.155 |
| Sleep irregularity and variability | 23.5% | 1.43 [0.87, 2.37] | 0.161 |  | 28.3% | 3.84 [1.53, 9.64] | **0.004** |  | 19.4% | 0.99 [0.50, 1.96] | 0.988 |
| Fragmented sleep | 18.5% | 1.12 [0.75, 1.67] | 0.567 |  | 20.3% | 2.62 [1.17, 5.85] | **0.019** |  | 17.0% | 0.83 [0.50, 1.37] | 0.458 |
| Prolonged sleep latency | 16.9% | 1.04 [0.52, 2.07] | 0.918 |  | 18.9% | 2.16 [0.72, 6.49] | 0.171 |  | 14.7% | 0.77 [0.28, 2.12] | 0.612 |
| Adjusted for sex (only for total population), age, study, study center, parental highest education, BMI measurement methods (only for adolescence), season, sedentary behavior, moderate-to-vigorous physical activity, and total energy intake. *P*-values < 0.05 were highlighted in bold. Abbreviations: BMI, body mass index; CI, confidence interval; OR, odds ratio. | | | | | | | | | | | |

| **Table S10. Associations of** **sleep clusters with BMI and overweight/obesity in participants with repeated two time-points data** | | | |
| --- | --- | --- | --- |
| **Sleep clusters** | **N=636, 1272 observations** | **Model** | |
| **BMI** | **Observations** | **β [95%CI]** | ***P*-value** |
| Good sleep | 441 | Ref |  |
| Delayed sleep phase | 176 | 0.07 [-0.24, 0.37] | 0.666 |
| Sleep irregularity and variability | 117 | 0.01 [-0.40, 0.41] | 0.973 |
| Fragmented sleep | 367 | 0.22 [-0.13, 0.58] | 0.211 |
| Prolonged sleep latency | 71 | 0.50 [-0.17, 1.18] | 0.140 |
| **Overweight/obesity** | **Cases/Observations** | **OR [95%CI]** | ***P*-value** |
| Good sleep | 15.4% | Ref |  |
| Delayed sleep phase | 16.3% | 1.05 [0.75, 1.46] | 0.795 |
| Sleep irregularity and variability | 18.8% | 1.12 [0.71, 1.77] | 0.616 |
| Fragmented sleep | 15.8% | 1.15 [0.81, 1.65] | 0.431 |
| Prolonged sleep latency | 22.5% | 1.78 [0.95, 3.35] | 0.074 |
| Adjusted for time of follow-ups, sex, age, study, study center, parental highest education, BMI measurement methods, season, sedentary behavior, moderate-to-vigorous physical activity, and total energy intake. Abbreviations: BMI, body mass index; CI, confidence interval; OR, odds ratio. | | | |

| **Table S11. Associations of sleep clusters with BMI and overweight/obesity, excluding participants with missing total energy intake information** | | | |
| --- | --- | --- | --- |
| **Sleep clusters** | **N=1630, 2082 observations** | **Model** | |
| **BMI** | **Observations** | **β [95%CI]** | ***P*-value** |
| Good sleep | 699 | Ref |  |
| Delayed sleep phase | 457 | 0.10 [-0.21, 0.41] | 0.540 |
| Sleep irregularity and variability | 189 | -0.09 [-0.50, 0.31] | 0.644 |
| Fragmented sleep | 598 | 0.26 [-0.08, 0.59] | 0.130 |
| Prolonged sleep latency | 139 | 0.29 [-0.28, 0.86] | 0.325 |
| **Overweight/obesity** | **Cases/Observations** | **OR [95%CI]** | ***P*-value** |
| Good sleep | 15.2% | Ref |  |
| Delayed sleep phase | 16.0% | 0.99 [0.74, 1.33] | 0.965 |
| Sleep irregularity and variability | 16.4% | 1.04 [0.70, 1.55] | 0.839 |
| Fragmented sleep | 17.2% | 1.23 [0.93, 1.64] | 0.151 |
| Prolonged sleep latency | 21.6% | 1.55 [0.95, 2.54] | 0.078 |
| Adjusted for time of follow-ups, sex, age, study, study center, parental highest education, BMI measurement methods, season, sedentary behavior, moderate-to-vigorous physical activity, and total energy intake. *P*-values < 0.05 were highlighted in bold. Abbreviations: BMI, body mass index; CI, confidence interval; OR, odds ratio. | | | |

| **Table S12. Associations of sleep clusters with BMI and overweight/obesity, excluding participants with parent-reported BMI** **in adolescence** | | | |
| --- | --- | --- | --- |
| **Sleep clusters** | **N=1890, 2459 observations** | **Model** | |
| **BMI** | **Observations** | **β [95%CI]** | ***P*-value** |
| Good sleep | 786 | Ref |  |
| Delayed sleep phase | 549 | 0.03 [-0.24, 0.30] | 0.824 |
| Sleep irregularity and variability | 251 | 0.12 [-0.22, 0.45] | 0.504 |
| Fragmented sleep | 704 | 0.30 [0.01, 0.59] | **0.041** |
| Prolonged sleep latency | 169 | 0.63 [0.10, 1.15] | **0.019** |
| **Overweight/obesity** | **Cases/Observations** | **OR [95%CI]** | ***P*-value** |
| Good sleep | 16.0% | Ref |  |
| Delayed sleep phase | 16.2% | 0.98 [0.75, 1.27] | 0.873 |
| Sleep irregularity and variability | 19.9% | 1.13 [0.80, 1.59] | 0.483 |
| Fragmented sleep | 18.5% | 1.23 [0.94, 1.59] | 0.127 |
| Prolonged sleep latency | 23.1% | 1.67 [1.11, 2.51] | **0.014** |
| Adjusted for time of follow-ups, sex, age, study, study center, parental highest education, season, sedentary behavior, moderate-to-vigorous physical activity, and total energy intake. *P*-values < 0.05 were highlighted in bold. Abbreviations: BMI, body mass index; CI, confidence interval; OR, odds ratio. | | | |

| **Table S13. Associations of BMI and overweight/obesity with sleep clusters identified by sleep characteristics, excluding the Friday night and Saturday night** | | | |
| --- | --- | --- | --- |
| **Sleep clusters** | **N=1973, 2609 observations** | **Model** | |
| **BMI** | **Observations** | **β [95%CI]** | ***P*-value** |
| Good sleep | 944 | Ref |  |
| Delayed sleep phase | 486 | 0.15 [-0.10, 0.39] | 0.244 |
| Sleep irregularity and variability | 249 | 0.33 [-0.04, 0.70] | 0.079 |
| Fragmented sleep | 729 | 0.27 [0.00, 0.53] | **0.047** |
| Prolonged sleep latency | 201 | 0.74 [0.28, 1.19] | **0.002** |
| **Overweight/obesity** | **Cases/Observations** | **OR [95%CI]** | ***P*-value** |
| Good sleep | 15.6% | Ref |  |
| Delayed sleep phase | 15.2% | 1.05 [0.80, 1.38] | 0.715 |
| Sleep irregularity and variability | 21.3% | 1.43 [1.03, 1.97] | **0.032** |
| Fragmented sleep | 17.6% | 1.20 [0.94, 1.55] | 0.148 |
| Prolonged sleep latency | 23.4% | 1.93 [1.34, 2.78] | **<0.001** |
| Adjusted for time of follow-ups, sex, age, study, study center, parental highest education, BMI measurement methods, season, sedentary behavior, moderate-to-vigorous physical activity, and total energy intake. *P*-values < 0.05 were highlighted in bold. Abbreviations: BMI, body mass index; CI, confidence interval; OR, odds ratio. | | | |

**
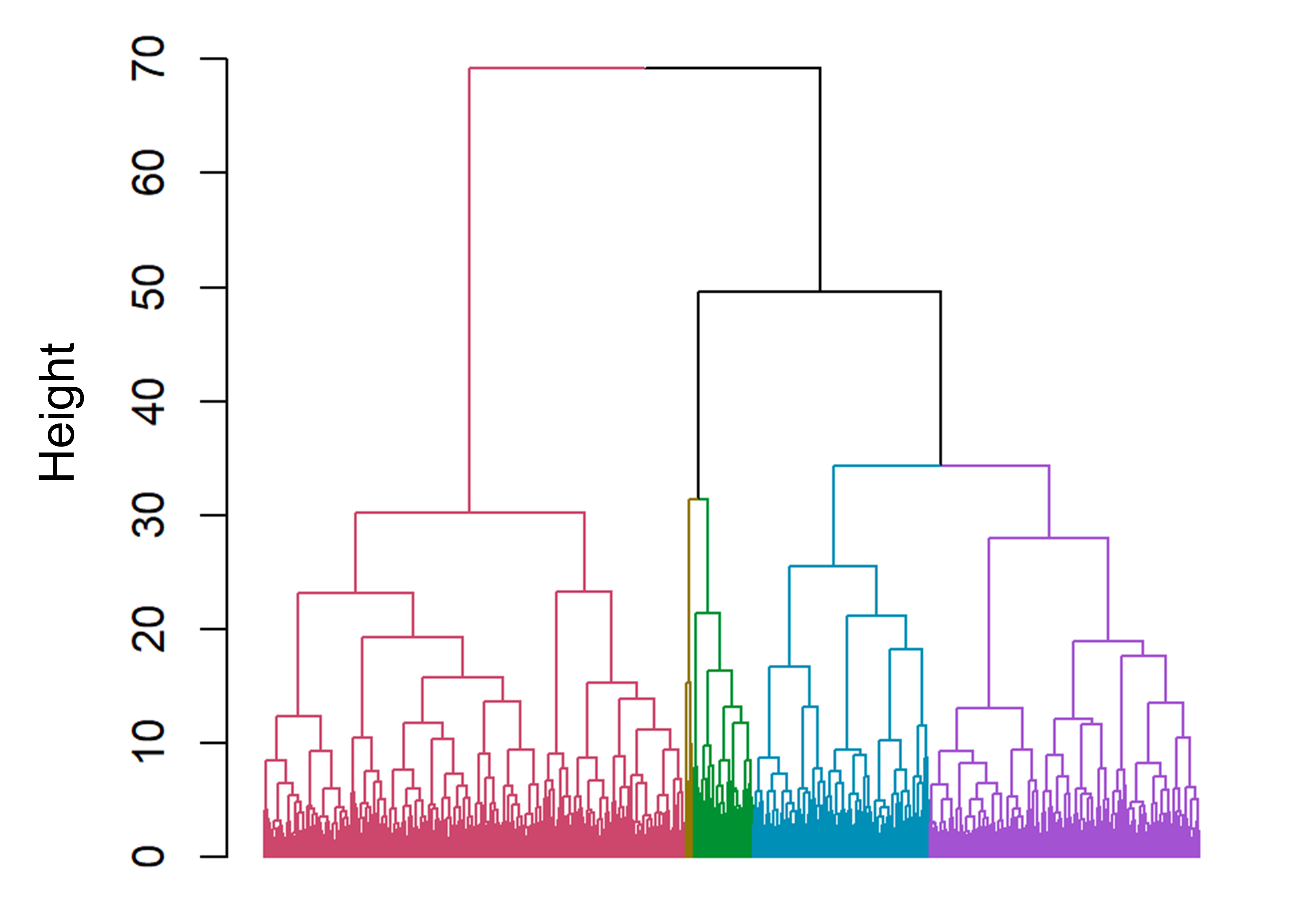
**

**Fig. S1.** Hierarchical clustering dendrogram based on 12 sleep characteristics from the 20-year data.

**Fig. S1** illustrates the dendrogram of the hierarchical clustering from the 20-year data, using Euclidean distance and Ward’s linkage across 12 standardized sleep characteristics. The y-axis of the dendrogram displays the “Height”, which represents the distance used to cluster the objects. In this agglomerative approach, each observation begins as its own cluster and is subsequently grouped with similar objects in the early stages. As the number of clusters decreases, the height increases, reflecting greater heterogeneity among clusters. Five different colors denote the final five clusters.

Note, the hierarchical clustering dendrogram based on 12 sleep characteristics from the 15-year data has been published previously [1].

**
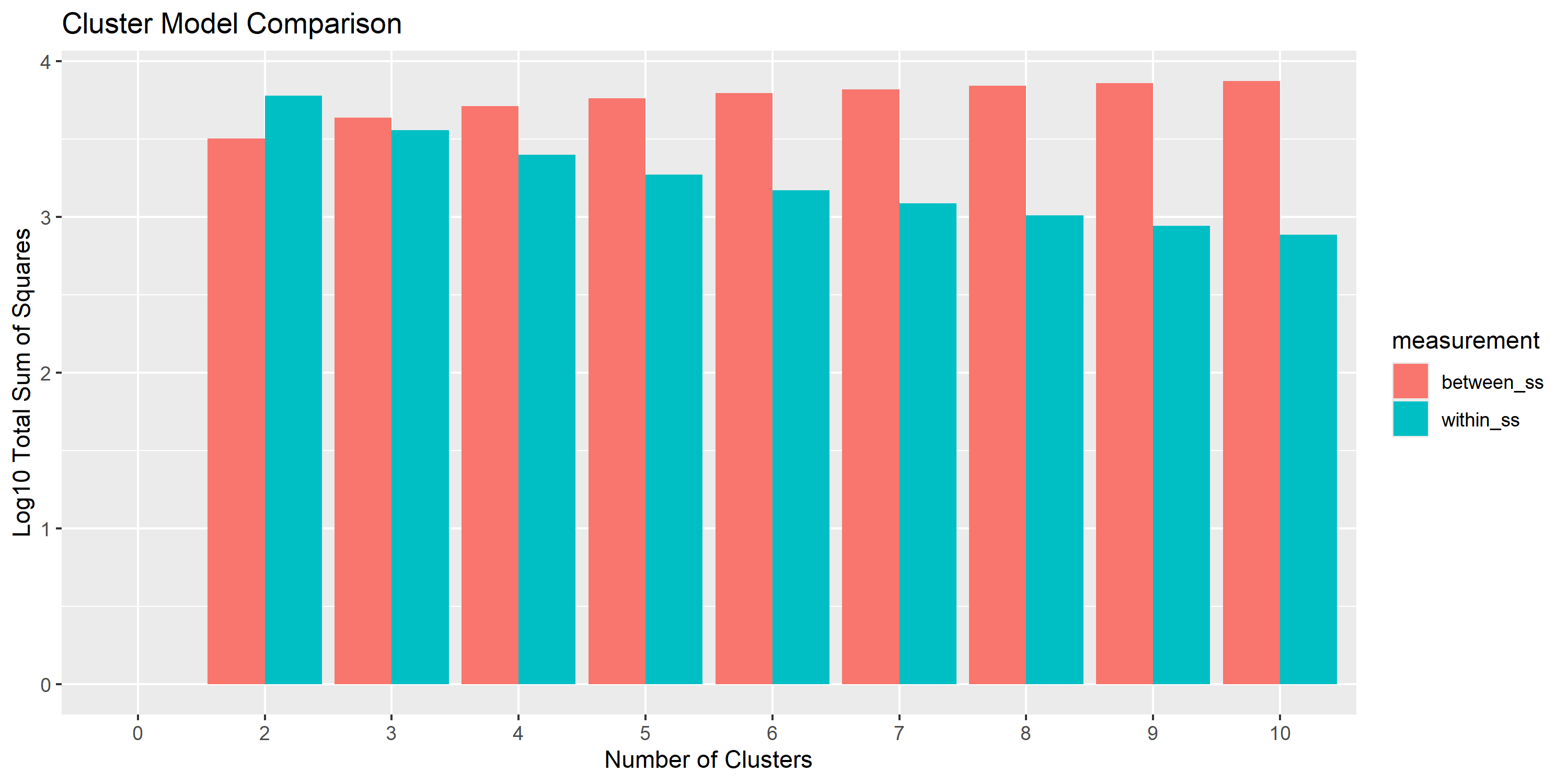
**

**Fig. S2.** Sum of squares method for k-means cluster model comparison from the 20-year data.

**Fig. S2** presents the visualization of the sum of squares method, which is used to determine the optimal number of clusters. This method evaluates the within-cluster sum of squares, representing cluster compactness (blue color, the smaller the better), and the between-cluster sum of squares, representing the separation among clusters (red color, the larger the better). The figure suggests that choosing five clusters is the appropriate option.

Note, you can find the sum of squares method for k-means cluster model comparison from the 15-year data in previous paper [1].

**
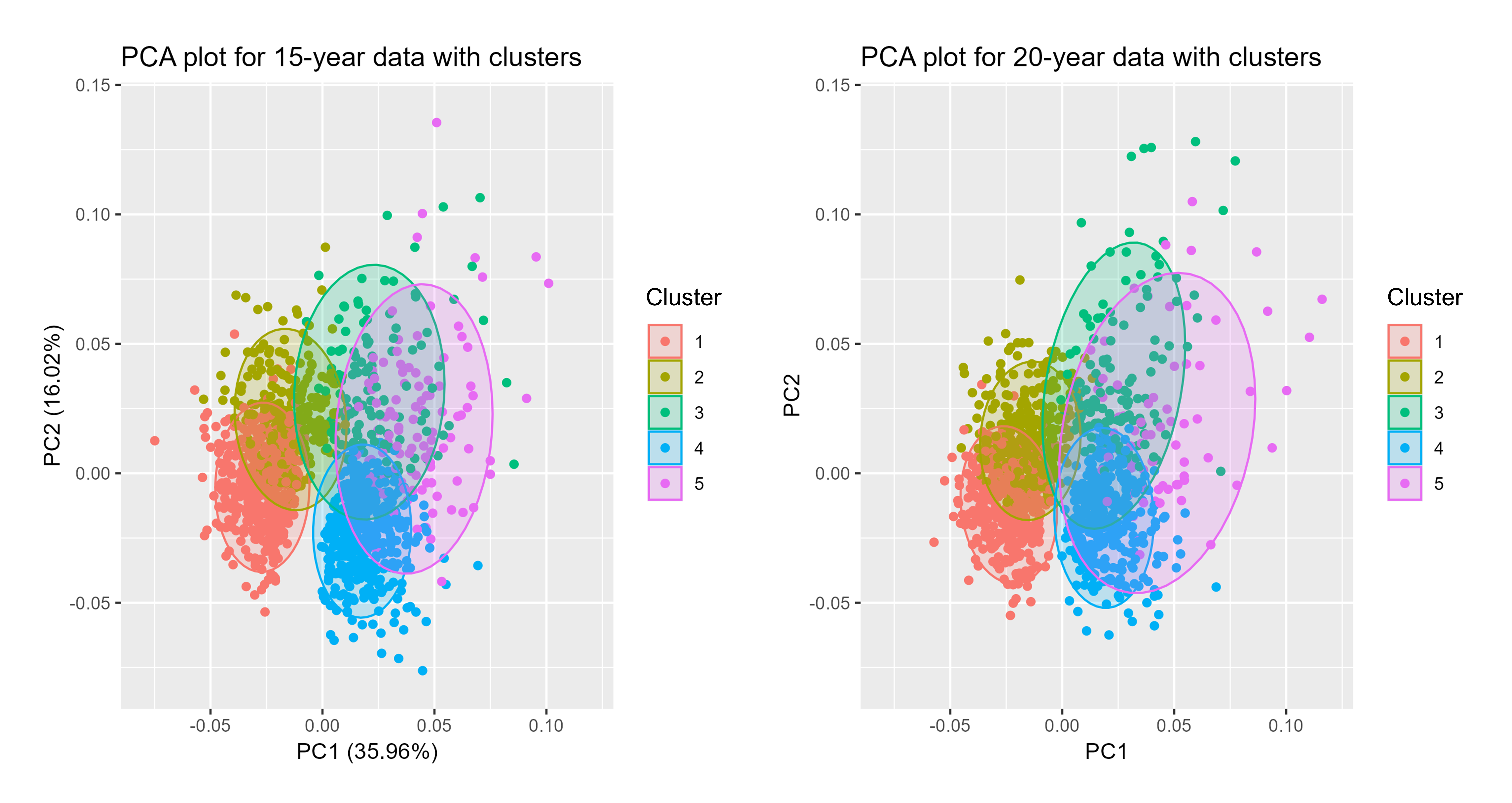
**

**Fig. S3.** Principal component analysis (PCA) plots for the validation of clustering results from 15-year data on 20-year data. The five sleep clusters at both data are: 1, “good sleep” cluster; 2, “delayed sleep phase” cluster; 3, “sleep irregularity and variability” cluster; 4, “fragmented sleep” cluster; 5, “prolonged sleep latency” cluster. The x-axis is the first principal component (PC), and the y-axis is the second PC.

In the **left plot** of **Fig. S3**, we display the 15-year data in the first two PCs, with each cluster represented by a distinct color, and ellipses drawn around the cluster centers. Cluster 1 (red) is distinctly separated from clusters 4 (blue) and 5 (purple) along the first PC. Clusters 1 (red), 2 (brown) and 3 (green) show some overlaps. There are some overlaps among Clusters 2 (brown), 3 (green), 4 (blue), and 5 (purple).

In the **right plot** of **Fig. S3**, we project the 20-year data onto the PCs of 15-year data via scaling and rotating, to compare more directly the PCA plot for 20-year data with the PCA plot for 15-year data (instead of calculating the PCA anew for the 20-year data). The distribution of 5 clusters is similar for both 15-year and 20-year data. For example, Cluster 1 (red) has a clear separation from Cluster 5 (purple) by the first PC, although there is small overlap with Cluster 4 (blue).

The validation of the PCA plots followed a published framework [2].

**References**

1. Wang M, Flexeder C, Harris CP, Thiering E, Koletzko S, Bauer CP, et al. Accelerometry-assessed sleep clusters and cardiometabolic risk factors in adolescents. Obesity (Silver Spring). 2024;32(1):200-13.

2. Ullmann T, Hennig C, Boulesteix AL. Validation of cluster analysis results on validation data: A systematic framework. WIREs Data Mining and Knowledge Discovery. 2021;12(3): e14444.
